# Limited generalizability and high risk of bias in multivariable models predicting conversion risk from mild cognitive impairment to dementia: A systematic review

**DOI:** 10.1002/alz.70069

**Published:** 2025-04-06

**Authors:** Robin Jeanna Vermeulen, Vebjørn Andersson, Jimmy Banken, Gerjon Hannink, Tim Martin Govers, Maroeska Mariet Rovers, Marcel Gerardus Maria Olde Rikkert

**Affiliations:** ^1^ Department of Medical Imaging Radboud University Medical Centre Nijmegen The Netherlands; ^2^ Department of Neurology Oslo University Hospital Oslo Norway; ^3^ Radboudumc Alzheimer Centre Department of Geriatrics Radboud University Medical Centre Nijmegen The Netherlands

**Keywords:** Alzheimer's disease, dementia, mild cognitive impairment, prediction model, risk prediction, systematic review

## Abstract

Prediction models have been developed to identify mild cognitive impairment (MCI) cases likely to convert to dementia. This systematic review summarizes multi‐source prediction models for MCI to dementia conversion. PubMed and Embase were searched for model development and validation studies from inception up to January 18 2024. Models were assessed for included predictors, predictive performance, risk of bias, and generalizability. 62 studies were included: 41 machine learning models, 11 regression models, and 5 disease state indexes. The number of predictors in the models ranged from 2 to 60; magnetic resonance imaging (MRI) and cognitive scores were the most common sources. Performance measures indicate reasonable predictive capabilities (area under the curve [AUC] range: 0.58–0.98, accuracy range: 66.1–96.3%); however, most studies are at high risk of bias and 47 studies lack external validation. Currently, no highly valid prediction model is available for MCI to dementia conversion risk due to limited generalizability and high risk of bias in most studies.

**Highlights:**

- Numerous models have been developed to predict the likelihood of conversion to dementia in individuals with MCI.
- Prediction models seem to have a reasonably good performance in predicting conversion to dementia, however, external validation and generalizability is often lacking.
- There is no prediction model available with a low risk for bias and that has been externally validated to accurately predict the risk of MCI to dementia conversion.
- For MCI to dementia conversion prediction models, more emphasis should be directed towards external validation, generalizability, and clinical applicability.

## 1 BACKGROUND

Dementia, currently affecting over 55 million individuals worldwide, is a leading cause of global disability. The symptoms of dementia, which only arise at a late disease state, lead to significant physical, psychological, social, and economic challenges, impacting not just the individuals diagnosed but also their caregivers and society at large.^1^ Predicting who will and who will not develop dementia may have considerable added value to mitigate the impact of the disease on both affected individuals and the broader society. Since the number of dementia cases is predicted to almost triple by 2050, the associated costs and burden are expected to increase drastically too.^2^, ^3^ This scenario underscores an urgent need for measures to prevent or delay disease progression, which are most likely to be effective in early or preclinical states of the disease.^4^, ^5^, ^6^ Yet, no effective preventive or curative treatment to stop the progression exists today.^3^, ^7^, ^8^

Mild cognitive impairment (MCI) is regarded as a potential target for interventions to delay progression to dementia, as it is a cognitive condition that signals most new dementia cases.^9^ MCI is characterized by cognitive deterioration that is significantly greater than expected for an individual's age, but not severe enough to interfere significantly with daily functioning.^10^ Although reported progression rates from MCI to dementia differ between research settings and populations, most frequently reported annual progression rates range from 10 to 15%.^11^ Both pharmacological and non‐pharmacological interventions have demonstrated some symptomatic benefit for individuals with MCI, albeit without preventing progression to dementia.^12^ Despite the current inability of early interventions to prevent dementia, the early and accurate identification of those at risk for progression to dementia is generally considered clinically relevant. Such identification is believed to be beneficial to target current and future interventions to those who would most likely benefit from it, to select cases for participation in clinical trials, and to start advanced care planning.

As a result, predictive factors for the progression from MCI to dementia have undergone extensive study, revealing several key predictors. These include the presence of specific biomarkers such as beta‐amyloid and tau aggregates in the brain, genetic predispositions such as apolipoprotein E (APOE) ε4 alleles, and medial temporal or global cortical atrophy.^13^ Many models, ranging from regression analyses to advanced machine learning algorithms integrating various types of predictors have been developed to predict dementia.^14^ These models aim to provide an accurate estimation of an individual's risk for converting from MCI to dementia. Combining predictors from different sources is believed to enhance the predictive performance over single‐source prediction models (e.g., demographics, image‐based features, and fluid biomarkers, etc).^15^, ^16^ This systematic review aims to provide an overview of multi‐source prediction models developed for predicting conversion from MCI to dementia and to evaluate their predictive performance. Additionally, through a comprehensive quality assessment, we aim to evaluate the strength of the evidence in the included prediction model studies and to indicate potential directions for the improvement of quality and reporting of future prediction model studies.

## 2 METHODS

This review is reported according to the Transparent Reporting of Multivariable Prediction Models for Individual Prognosis or Diagnosis Tailored for Systematic Reviews and Meta‐analyses (TRIPOD‐SRMA) guideline.^17^

### 2.1 Search strategy

We systematically searched the databases PubMed and Embase for studies reporting on the development and/or validation of a prediction model for MCI to dementia conversion. We searched for studies that were published from inception to January 18 2024. The search query was constructed in consultation with a librarian of the Radboud University Medical Center and was a combination of the following terms and their synonyms: “mild cognitive impairment”, “dementia”, “prognostic model”, and “performance”. Search terms were used as keywords in title and abstract, and, if available, in combination as MeSH terms to maximize the output from literature findings. The exact search strategy for both databases is shown in the supplementary material (Table S1). Only articles published in the English language were considered for review. In order to identify any additional papers that might be relevant for inclusion in this review, backward and forward reference searching of eligible studies was performed using Citationchaser.^18^

### 2.2 Eligibility criteria

Studies were included if they reported on a multivariable prediction model for conversion to dementia over a minimal prediction horizon of 2 years. This is a clinically relevant prediction horizon since conversion from MCI to dementia most commonly happens within a period of 3 to 5 years.

To determine the eligibility for inclusion in this review studies were assessed according to the following inclusion criteria: (1) inclusion of people with MCI; (2) model for predicting conversion to dementia among people with MCI; (3) prediction model based on combination of predictors from multiple sources (e.g., demographics, imaging‐based features, and cognitive test scores); (4) aimed at individual risk prediction; (5) model performance evaluated. Exclusion criteria were: (1) abstract only; (2) conference abstract; (3) poster presentation; (4) editorial; (5) case report; (6) review article; (7) prediction of dementia in a patient population with underlying diseases that elevate the risk on dementia (e.g., Parkinson's disease); (8) prediction of dementia in a subgroup of MCI cases (e.g., APOE4 positive); (9) prediction horizon less than two years; (10) less than 100 participants included for model development; (11) methodological papers using dementia as a use case; (12) no internal validation step; (13) multi‐source predictor model is not better than single source predictor model developed in the same study; (14) unclear which candidate predictors are included in the final model. Both development and validation studies, or a combination of both, were eligible for inclusion in this review. Two researchers (R.V., V.A.) independently screened the title and abstracts of the retrieved studies for possible inclusion based on the criteria mentioned above. Full text papers of the retained studies were evaluated for final inclusion by the authors. In case of discrepancies a third reviewer was involved (T.G.). The Rayyan AI support tool was used during the study selection process.^19^

### 2.3 Quality assessment

The quality of the included studies was evaluated separately by two authors per study (R.V., V.A., J.B.). Any discrepancies were resolved by a third investigator (R.V., V.A., J.B.) or (T.G.). Quality control included assessment of risk of bias and applicability using the PROBAST tool, which is specifically designed for the assessment of risk of bias and applicability of prediction model studies.^20^, ^21^ The PROBAST tool assesses the risk of bias of prediction model studies across four domains: selection of participants, predictors and their assessment, outcome and its determination, and analysis. The applicability of the models for answering the review question is also assessed for the first three domains of the PROBAST: Do included participants or setting match the review question?; Do definition, assessment, or timing of predictors match the review question?; Do definition, timing, or determination of the outcome match the review question?

**RESEARCH IN CONTEXT**

1. **Systematic review**: This review provides a comprehensive overview of multivariable prediction models for conversion from mild cognitive impairment (MCI) to dementia. Evaluation focused on included predictors, predictive performance, risk of bias and generalizability.
2. **Interpretation**: Numerous models have been developed to predict conversion from MCI to dementia, and their predictive performance appears to be reasonably good. However, most lack external validation, limiting generalizability to diverse patient populations. Moreover, models often rely on a wide variety of predictors, some of which are not easily and readily accessible, which potentially hinders their clinical applicability. The significant number of identified studies reflects the growing emphasis on early identification of individuals at risk for dementia.
3. **Future directions**: Future research should prioritize development and validation of robust prediction models, with improved generalizability and clinical utility. This involves not only enhancing the performance of these models but also ensuring their applicability to various populations and healthcare environments.

### 2.4 Data extraction

The CHARMS checklist was used to collect required data from the included studies.^22^ For each included study, two reviewers independently extracted the data (R.V., V.A., J.B.). The following information was extracted from the studies: first author, year of publication, source of data, date or period of obtaining data, subjects included, type of dementia, MCI diagnostic criteria, dementia diagnostic criteria, sample size, dementia incidence, model development method, model validation method, type of classification (e.g., converter vs. stable MCI, risk categories such as low, intermediate, high risk of conversion), mean follow‐up time, prediction horizon, predictor sources (e.g., MRI, fluid biomarkers) and predictors extracted from the sources (e.g., hippocampal volume, amyloid beta), time of predictor measurements (baseline/longitudinal data), any model performance measures provided.

If a study tested multiple predictor combinations or model development methods, we report the model with the combination of predictors or the model type that yielded the best predictive performance as reported in the article. If a model was developed to make predictions for different prediction horizons, we report the results of the prediction horizon that showed the best predictive performance as reported in the article. Nevertheless, the prediction horizon should at least be 2 years from the moment of prediction until performance of the reference diagnosis. Studies were grouped in three categories: model development studies (development and internal validation of a prediction model), model development and validation studies (development and external validation of a prediction model), and model validation studies (external validation of a prediction model). In the context of external validation, a model is tested on a different cohort of participants. Studies that developed a classification model in people with Alzheimer's disease (AD) and cognitively normal (CN) people and subsequently tested the model for predicting converter MCI versus stable MCI were categorized as model development and validation studies.

## 3 RESULTS

### 3.1 Literature search

**FIGURE 1:**
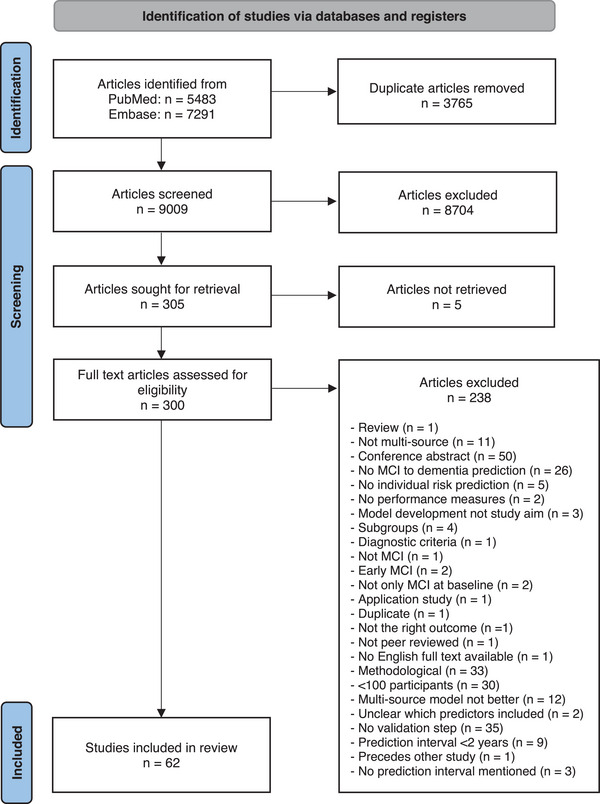
Flowchart of the study selection process.

Figure 1 shows the results of literature search and selection. A total of 12,774 articles were retrieved, of which 9009 remained after removing duplicates. Based on title and abstract screening, 8704 articles were excluded. Of the 305 studies eligible for full text screening, 5 articles could not be retrieved. After full text screening, 62 articles remained eligible for inclusion in this review.

### 3.2 Description of included studies

The study characteristics of the individual studies are summarized in Table 1. Of the 62 included studies, 47 only developed a model for predicting conversion from MCI to dementia, 11 developed a model which was also externally validated, and in 4 studies a previously developed prediction model was externally validated. All studies were cohort studies, and most of the studies (*n* = 55, 89%) used the Alzheimer's Disease Neuroimaging Initiative (ADNI) dataset for the development or validation of a prediction model. Other cohorts that are used were the DESCRIPA cohort (*n* = 2), the CREDOS cohort (*n* = 1), the Amsterdam Dementia Cohort (ADC) (*n* = 3), Gothenburg MCI study (*n* = 1), Kuopio MCI cohort (*n* = 1), AddNeuroMed cohort (*n* = 1), QD cohort (*n* = 1), EMIF‐AD cohort (*n* = 1), Swedish BioFINDER study (*n* = 1), National Alzheimer's Coordinating Center (NACC) database (*n* = 1), Japanese‐ADNI (*n* = 1), or cohorts of participants that were included at specific medical centers (*n* = 4). Sample sizes ranged from 126 to 2448 in the model development studies, from 143 to 1329 in the model development and validation studies, and from 211 to 2611 in the model validation studies. MCI was defined according to the Petersen criteria (*n* = 7), NIA‐AA criteria (*n* = 5), Mayo clinic criteria (*n* = 1), a *z*‐score of ≤1.5 in neuropsychological tests (*n* = 3), NACC criteria (*n* = 1), AddNeuroMed criteria (*n* = 1), or according the specifically defined ADNI MCI criteria (*n* = 55). See Table S2 for a description of the different criteria. Dementia incidence ranged from 16.8% to 65.6% in the model development studies, from 19% to 57% in the model development and validation studies, and from 26.1% to 65.9% in the model validation studies. Thirty‐eight studies reported the criteria for dementia, and most of these used the NINCDS‐ADRDA criteria for probable AD (*n* = 36).^23^ Studies that not explicitly described criteria for dementia referred to the study protocol, which was ADNI for most of the studies.^24^ In 56 of the studies, conversion to AD type dementia was predicted, whereas in only 6 studies the model was developed/validated for predicting the conversion to any type of dementia.

**TABLE 1:** Study characteristics

Study	Source of data	Date obtaining data	Subjects included	Type of dementia	MCI criteria	Dementia assessment	Sample size	Dementia incidence
**Model development studies (development + internal validation)**								
Adelson 2023^26^	ADNI	NI	MCI	AD	ADNI	MMSE < 27, CDR 0.5 or 1.0, Logical Memory Scale‐Revides score up to 25	493 MCI	52.5%
Ardekani 2016^27^	ADNI	October 2013	MCI	AD	ADNI	MMSE scores between 20‐26 (inclusive), CDR of 0.5 or 1.0 and NINCDS‐ADRDA for probable AD	164 MCI	52.4%
Bapat 2024^28^	ADNI	NI	MCI	AD	ADNI	NI	215 MCI	40%
Barnes 2014^29^	ADNI	July 2012	MCI	AD	ADNI	NINCDS‐ADRDA for probable AD	382 MCI	46.9%
Blazhenets 2020^30^	ADNI	NI	MCI	AD	ADNI	NI	319 MCI	22.6%
Bouallègue 2017^31^	ADNI	NI	CN, MCI, AD	AD	ADNI	NINCDS‐ADRDA for probable AD	251 CN 289 MCI 144 AD	29.4%
Cai 2023^32^	ADNI	October 2022	MCI	AD	ADNI	NINCDS‐ADRDA for probable AD	1042 MCI	37.8%
Cao 2023^33^	ADNI	NI	CN, MCI, AD	Any type dementia	ADNI	NI	150 CN 257 MCI 205 AD	36.2%
Chang 2022^34^	ADNI	NI	MCI	AD	ADNI	NINCDS‐ADRDA for probable AD	160 MCI	42.5%
Chun 2022^35^	Samsung Medical Center (SMC), South Korea	Inclusion June 2007 ‐ December 2019	MCI	Any type dementia	NIA‐AA	DSM‐IV and NINCDS‐ADRDA for probable AD	705 MCI	36.1%
Devenand 2008^36^	Memory Disorders Clinic New York State Psychiatric Institute/Columbia University	NI	MCI	AD	Petersen	DSM‐IV and NINCDS‐ADRDA for probable AD	126 MCI	26.2%
Dobromsylin 2022^37^	ADNI	NI	MCI	AD	ADNI	NI	232 MCI	16.8%
El‐Sappagh 2021^38^	ADNI	NI	CN, MCI, AD	AD	ADNI	NINCDS‐ADRDA for probable AD	294 CN 486 MCI 268 AD	47.7%
Franciotti 2023^39^	ADNI	NI	MCI	AD	ADNI	NI	587 MCI	40.2%
Goel 2023^40^	ADNI	NI	CN, MCI, AD	AD	ADNI	NI	883 CN 1132 MCI 433 AD	NI
Grassi 2019^41^	ADNI	NI	MCI	AD	ADNI	MMSE scores between 20–26 and NINCDS‐ADRDA for probable AD	550 MCI	35.8%
Hall 2015^42^	DESCRIPA	NI	SCI, MCI	AD	*z*‐score ≤1.5 in neuropsycho‐logical tests	NINCDS‐ADRDA for probable AD	544 MCI	27.4%
Hou 2023^43^	ADNI	NI	MCI	AD	ADNI	NI	796 MCI	40.3%
Jang 2017^44^	Samsung Medical Center (SMC) & Clinical Research Center for Dementia of South Korea (CREDOS)	SMC: Inclusion June 2007 ‐ December 2011. CREDOS: Inclusion November 2005 ‐ December 2011.	MCI	Any type dementia	Petersen	DSM‐IV and NINCDS‐ADRDA for probable AD	242 MCI	61.5%
Kauppi 2018^45^	ADNI	November 2011	MCI	AD	ADNI	NI	336 MCI	54.2%
Khajehpiri 2022^46^	ADNI	NI	MCI	AD	ADNI	MMSE scores between 20–26 (inclusive), CDR of 0.5 or 1.0 and NINCDS‐ADRDA for probable AD	882 MCI	39.2%
Korolev 2016^47^	ADNI	Clinical data: August 2011; Structural MRI data: August 2011; Plasma proteomic data: June 2012	MCI	AD	ADNI	NINCDS‐ADRDA for probable AD	289 MCI	53.7%
Lee 2014^48^	ADNI	July 2012	MCI	AD	ADNI	NINCDS‐ADRDA for probable AD	382 MCI	46.9%
Lee 2019^49^	ADNI	NI	CN, MCI, AD	AD	ADNI	NI	415 CN 865 MCI 338 AD	35.5%
Luk 2018^50^	ADNI	July 2016	CN, MCI, AD	AD	ADNI	MMSE scores between 20–26 (inclusive), CDR of 0.5 or 1.0 and NINCDS‐ADRDA for probable AD	204 MCI	48.0%
Mattila 2012^51^	ADNI	September 2011	MCI	AD	ADNI	NINCDS‐ADRDA for probable AD	288 MCI	48.6%
Mubeen 2017^52^	ADNI	NI	MCI	AD	ADNI	MMSE scores between 20–26 (inclusive), CDR of 0.5 or 1.0 and NINCDS‐ADRDA for probable AD	247 MCI	65.6%
Munoz‐Ruiz 2014^53^	ADNI	NI	MCI	AD	ADNI	NI	376 MCI	43.6%
Pang 2023^54^	NACC (National Alzheimer's Coordinating Center), United States	March 2021	MCI	AD	NACC	NI	882 MCI	36.4%
Park 2022^55^	J‐ADNI	May 2017	MCI	Any type dementia	ADNI	CDR ≥0.5 and NINCDS‐ADRDA for probable AD	179 MCI	54.8%
Peng 2023^56^	ADNI	NI	MCI	AD	ADNI	NI	341 MCI	29.9%
Platero 2020^57^	ADNI	NI	MCI	AD	ADNI	NI	321 MCI	48.6%
Platero 2021^58^	ADNI	NI	CN, MCI, AD	AD	ADNI	NI	352 MCI	48.9%
Runtti 2014^59^	ADNI	September 2011	MCI	AD	ADNI	NINCDS‐ADRDA for probable AD	289 MCI	48.4%
Shu 2021^60^	ADNI	NI	MCI	AD	ADNI	NI	357 MCI	43.1%
Tabatabaei‐Jafari 2018^61^	ADNI	NI	CN, MCI	AD	ADNI	MMSE scores less than 26, CDR of 0.5 or 1.0 and NINCDS‐ADRDA for probable AD	338 MCI	55.3%
Tam 2019^62^	ADNI	NI	CN, MCI, AD	AD	ADNI	NI	393 CN 470 MCI 254 AD	43.0%
Tang 2021^63^	ADNI	NI	MCI	AD	ADNI	NINCDS‐ADRDA for probable AD	162 MCI	41.9%
Varatharajah 2019^64^	ADNI	NI	MCI	AD	ADNI	NI	135 MCI	28.9%
Wang 2016^65^	ADNI	NI	MCI	AD	ADNI	NI	129 MCI	49.6%
Wang 2023^66^	ADNI	NI	MCI	Any type dementia	ADNI	NINCDS‐ADRDA for probable AD	365 MCI	38.9%
Willette 2014^67^	ADNI	October ‐November 2012	CN, MCI, AD	AD	ADNI	MMSE scores between 20–26 (inclusive), CDR of 0.5 or 1.0 and NINCDS‐ADRDA for probable AD	162 MCI	46.9%
Wu 2023^68^	ADNI	NI	MCI	AD	ADNI	NI	627 MCI	42.9%
Xu 2016^69^	ADNI	NI	CN, MCI	AD	ADNI	NI	117 CN 110 MCI	24.5%
Yang 2012^70^	ADNI	NI	CN, MCI, AD	AD	ADNI	MMSE scores between 20–26 (inclusive), CDR of 0.5 or 1.0 and NINCDS‐ADRDA for probable AD	72 CN 111 MCI 35 AD	22.5%
Ye 2012^71^	ADNI	August 2010	MCI	AD	ADNI	NI	319	44.5%
Zandifar 2020^72^	ADNI	NI	MCI	AD	ADNI	NINCDS–ADRDA for probable AD	499 MCI	34.9%
**Model development and validation studies (development + external validation)**								
Cui 2011^73^	ADNI	NI	CN, AD, MCI	AD	ADNI	MMSE scores between 20–26 (inclusive), CDR of 0.5 or 1.0 and NINCDS‐ADRDA for probable AD	143 MCI	39.2%
Dukart 2015^74^	ADNI	December 2013	CN, MCI, AD	AD	ADNI	NINCDS‐ADRDA for probable AD	112 CN 144 AD 442 MCI	40.0%
Ezzati 2019^75^	ADNI	September 2018	CN, MCI, AD	AD	ADNI	MMSE scores between 20–26 (inclusive), CDR of 0.5 or 1.0 and NINCDS‐ADRDA for probable AD	424 CN 656 MCI 249 AD	35.8%
Hall 2015^76^	ADNI, AddNeuroMed, DESCRIPA, Kuopio MCI cohort	NI	MCI	AD	ADNI AddNeuroMed DESCRIPA: *z*‐score ≤1.5 in neuropsycholo‐gical tests Kuopio: Mayo clinic criteria	ADNI: NI AddNeuroMed: NINCDS‐ADRDA for probable AD DESCRIPA: NINCDS‐ADRDA for probable AD Kuopio MCI cohort: NINCDS‐ADRDA for probable AD	ADNI: 370 MCI DESCRIPA: 237 MCI AddNeuroMed: 123 MCI Kuopio MCI: 145 MCI	ADNI: 44% DESCRIPA: 24% AddNeuroMed: 19% Kuopio MCI cohort: 37%
Kruczyk 2012^77^	Malmo University Hospital, Sweden & Gothenburg MCI study at memory Clinic at Sahlgrenska University Hospital, Mölndal	NI	CN, MCI	AD	Petersen	DSM‐IIIR and NINCDS‐ADRDA for probable AD	Training set: 39 CN 134 MCI Test set: 17 NC 83 MCI	Malmo University Hospital: 42.5% Gothenburg MCI study: NI
Ning 2018^78^	ADNI	NI	CN, MCI, AD	AD	ADNI	NI	225 CN 358 MCI 138 AD	46.4%
Tong 2017^79^	ADNI	NI	CN, MCI, AD	AD	ADNI	NI	229 CN 300 MCI 191 AD	57%
Van Maurik 2017^80^	ADC ADNI	ADC: Inclusion September 1997 ‐ August 2014 ADNI: NI	MCI	Any type dementia	ADC: before 2012 Petersen, from 2012 NIA‐AA ADNI	AD: NINCDS‐ADRDA for probable AD Other dementia: established clinical criteria	Training set: 525 MCI External validation set: 295 MCI	48.2%
Van Maurik 2019^81^	ADNI ADC	NI	MCI	AD	ADNI ADC: before 2012 Petersen, from 2012 NIA‐AA	NINCDS‐ADRDA for probable AD	Development cohort: 411 MCI Validation cohort: 107 MCI	ADNI: 24.3% ADC: 29.0%
Westman 2012^82^	ADNI	NI	CN, MCI, AD	AD	ADNI	MMSE scores between 20‐26, CDR of 0.5 or 1.0, NINCDS/ADRDA criteria for probable AD, GDS b6	111 CN 162 MCI 96 AD	50%
Young 2013^83^	ADNI	NI	CN, MCI, AD	AD	ADNI	MMSE scores between 20‐26, CDR of 0.5 or 1.0, and NINCDS‐ADRDA criteria for probable AD	73 CN 143 MCI 63 AD	32.9%
**Model validation studies (external validation)**								
Devenand 2012^84^	ADNI, QD	ADNI: October 2010 QD: NI	MCI	AD	ADNI QD: z‐score ≤1.5 in neuropsycho‐logical tests	QD: NI ADNI: NI	282 MCI	QD: 26.1% ADNI: 55.6%
Liu 2013^85^	ADNI	NI	MCI	AD	ADNI	NI	391 MCI	40%
Rhodius‐Meester 2016^86^	ADC	NI	MCI	AD	Petersen and NIA‐AA	NINCDS‐ADRDA for probable AD	211 MCI	65.9%
van Maurik 2019^a^ ^,^ ^87^	EMIF‐AD, ADNI, ADC, Swedish BioFINDER study	NI	MCI	Any type dementia	EMIF‐AD: different criteria per cohort^b^ ADNI ADC: Petersen and NIA‐AA BioFINDER: MMSE 24‐30, does not fulfil criteria for dementia	NI	2611 MCI	EMIF‐AD: 32.7% ADNI: 38.5% ADC: 43.2% BioFINDER: 54.9%

Abbreviations: AD, Alzheimer's disease; ADC, Amsterdam Dementia Cohort; ADNI, Alzheimer's Disease Neuroimaging Initiative; CDR, clinical dementia rating scale; CN, cognitively normal (some studies use another term for cognitively normal, see Table 2); DSM, Diagnostic and Statistical Manual of Mental Disorders; EMIF‐AD, European Medical Information Framework for Alzheimer's Disease; GDS, Geriatric Depression Scale; MCI, mild cognitive impairment; MMSE, Mini‐Mental State Examination; NI, no information; NIA‐AA, National Institute on Aging and Alzheimer's Association; NINCDS‐ADRDA, National Institute of Neurological and Communicative Disorders and Stroke and the Alzheimer's Disease and Related Disorders Association; QD,; SCI, subjective cognitive impairment.

^a^ In this study also a new updated model is proposed based on the same cohorts (model development + external validation).

^b^ See supplementary material Van Maurik 2019.^1^

### 3.3 Description of the prediction models

The characteristics of the prediction models are summarized in Table 2. Seven of the prediction models were developed as a classification model in AD and CN cases. These models were subsequently validated for predicting conversion from MCI to dementia in people with MCI. Fifty‐five of the prediction models were both developed and validated in MCI subjects (internal or external validation). In one of the studies, the prediction model aimed to divide MCI cases into risk groups (e.g., low, intermediate, high risk of conversion to dementia), whereas in the other studies, the model aimed to distinguish between cases that convert to dementia over time versus cases that remain stable. Different terms are used to describe cases who convert to dementia over time or that remain stable (see Table S3), but all evaluate whether cases that were diagnosed with MCI at baseline are diagnosed with dementia somewhere within the prediction horizon or not. In this paper, we use the terms stable MCI and converter MCI for people that remain stable or people that develop dementia within the prediction horizon. The prediction horizons ranged from 2 to 8 years, with most studies predicting conversion within a period of 2 or 3 years (*n* = 55, 89%). Predictors in the models could be categorized into seven predictor sources: demographics, (family) history, genetics, cognitive test scores, MRI, positron emission tomography (PET), and fluid biomarkers (e.g., Aβeta, tau) measured in blood or cerebrospinal fluid (CSF). MRI used in the prediction models comprised image derived phenotypes extracted from MR images.^25^ In Table 3, the predictor sources that were used in each of the dementia risk prediction models are shown. The most commonly used predictors in the models were derived from MRI and cognitive tests, followed by genetics and demographics, fluid biomarkers, and PET. In the 62 included studies, 27 unique combinations of predictor sources were used. A combination of MRI measures and cognitive test scores was most frequently used (in 10 models), followed by a combination of demographics, genetics, cognitive scores, and MRI (in nine models). The number of candidate predictors considered for inclusion in the prediction models ranged from 3 to > 1.8 million. The latter was based on the extraction of over 1.8 million voxel features from MR images. Many different predictor selection strategies were used to select predictors to be included in the models (Table S4). A large variety of predictors was extracted from each of the sources to construct the different prediction models. Figure S1 shows the different predictors that were extracted per source in each of the models. The number of predictors included in the final models ranged from 2 to 60. Predictors that were included in at least 10 of the models are: age, sex, education, APOE4, Trail Making Test, MMSE, CDR‐SB, ADAS‐COG11, ADAS‐COG13, clock test score, FAQ score, RAVLT, Ttau, Amyloid Beta, hippocampal measures, and entorhinal cortex measures. Any form of internal validation (e.g., bootstrap, cross‐validation) was performed in 44 of the studies, 7 of the studies only randomly divided the participant sample in a training and test set, and 11 of the studies performed external validation of the model in a different cohort of participants. The models that are described in the included studies can be characterized as: machine learning models (*n* = 41), regression models (i.e., logistic or Cox regression) (*n* = 11), and disease state indexes (*n* = 5). In nine of the studies, a measure for model calibration was provided: seven presented a calibration curve, one reported a concordance correlation coefficient (CCC), and one provided an integrated calibration index (ICI). In 38 of the development studies, in 10 of the development and validation studies, and in 3 of the validation studies, a measure for model discrimination (AUC, Harrell's C) was provided. Classification accuracy was reported in 36 of the development studies, 7 of the development and validation studies, and 2 of the validation studies. The calibration curves in the seven studies showed good agreement between the observed and predicted outcomes according to the authors, the CCC in the study of Korolev 2016 was 0.95, and the ICI in the study of Wang 2023 was 0.06. The AUC/Harrell's C ranged from 0.71 to 0.98 in the development studies, from 0.58 to 0.92 in the development and validation studies, and from 0.74 to 0.87 in the validation studies. The (balanced) accuracy ranged from 66.7% to 96.3% in the development studies, from 66.1% to 87% in the development and validation studies, and from 71% to 77% in the validation studies. Other performance measures are reported in Table S4.

**TABLE 2:** Model characteristics

Study	Model type	Model validation	Type of classification	Follow‐up time mean (SD)	Baseline/longitudinal data^a^	Prediction horizon	Model performance
**Model development studies (development + internal validation)**							
Adelson 2023^26^	Gradient‐boosted tree ensemble (XGboost)	Bootstrapping	Stable MCI versus Converter MCI	NI	Baseline	2 years	AUC = 0.98 ACC = 88.9%
Ardekani 2016^27^	Random forest	Bootstrapping	Stable MCI versus Converter MCI	NI	Baseline	3 years	AUC = 0.77 ACC = 72.6%
					Longitudinal: 1 year	2 years	AUC = 0.83 ACC = 82.3%
Bapat 2024^28^	Convolutional neural network	5‐Fold cross‐validation	Stable MCI versus Converter MCI	NI	Baseline	4 years	AUC = 0.864 Balanced ACC = 79.9%
					Longitudinal: 1.5 year	2.5 years	AUC = 0.915 Balanced ACC = 81.0%
Barnes 2014^29^	Cox proportional hazards model	Bootstrapping	Stable MCI versus Converter MCI	Converter MCI: 2.9 (1.1)	Baseline	3 years	Harrell's C = 0.78 CC = good calibration
Blazhenets 2020^30^	Cox proportional hazards model	Random split in development and validation set	Low, medium, and high risk group	Development set: median = 48^36^, ^37^, ^38^, ^39^, ^40^, ^41^, ^42^, ^43^, ^44^, ^45^, ^46^, ^47^, ^48^, ^49^, ^50^, ^51^, ^52^, ^53^, ^54^, ^55^, ^56^, ^57^, ^58^, ^59^, ^60^, ^61^ months Validation set: median = 47^35^, ^36^, ^37^, ^38^, ^39^, ^40^, ^41^, ^42^, ^43^, ^44^, ^45^, ^46^, ^47^, ^48^, ^49^, ^50^, ^51^, ^52^ months	Baseline	During follow‐up	Harrel's C = 0.87^b^
Bouallègue 2017^31^	Support vector machine	Leave‐one‐out cross‐validation	Stable MCI versus Converter MCI	37^14^ months	Baseline	During follow‐up	ACC = 77%
Cai 2023^32^	Explainable boosting machine	5‐Fold cross‐validation	Stable MCI versus Converter MCI	NI	Baseline	5 years	AUC = 0.92
Cao 2023^33^	Convolutional neural network	5‐Fold cross‐validation	Stable MCI versus Converter MCI	NI	Baseline	3 years	AUC = 0.785 Balanced ACC = 73.3%
Chang 2022^34^	Logistic regression	Leave‐one‐out cross‐validation	Stable MCI versus Converter MCI	NI	Baseline	2 years	AUC = 0.75 ACC = 68%
Chun 2022^35^	Extreme gradient boost	5‐Fold cross‐validation	Stable MCI versus Converter MCI	NI	Baseline	3 years	AUC = 0.85 ACC = 80.7%
Devenand 2008^36^	Logistic regression analysis	5‐Fold cross‐validation	Stable MCI versus Converter MCI	Converter MCI: 41.5 (18.5) months Stable MCI: 57.3 (28.3) months	Baseline	3 years	AUC = 0.95 ACC = 89.7%
Dobromsylin 2022^37^	Logistic regression model	5‐Fold cross‐validation	Stable MCI versus Converter MCI	NI	Baseline	2 years	AUC = 0.80
El‐Sappagh 2021^38^	Random forest	10‐Fold cross‐validation	Stable MCI versus Converter MCI	NI	Baseline	3 years	AUC = 0.87 ACC = 87.1%
Franciotti 2023^39^	eXtreme Gradient Boosting^c^	Random split in train and test set	Stable MCI versus converter MCI	NI	Baseline	3 years	ACC = 0.89
Goel 2023^40^	Random forest	10‐Fold cross‐validation	Stable MCI versus converter MCI	NI	Baseline	2 years	AUC = 0.90 Balanced accuracy = 89.4%
Grassi 2019^41^	Weighted rank of multiple machine learning methods	10‐Fold cross‐validation	Stable MCI versus Converter MCI	NI	Baseline	3 years	AUC = 0.88 Balanced ACC = 0.79
Hall 2015^42^	Disease state index	Leave‐one‐out cross‐validation	Stable MCI versus Converter MCI	2.5 (0.9) years	Baseline	During follow‐up	AUC = 0.77 ACC = 0.71
Hou 2023^43^	Least absolute shrinkage and selection operator cox regression	Random split in discovery and validation cohort	Stable MCI versus Converter MCI	4.6 years	Baseline	5 years	AUC = 0.92
Jang 2017^44^	Logistic regression analysis	Bootstrapping, 10‐Fold cross‐validation, and random split in training and testing cohort	Stable MCI versus Converter MCI	2.92^2^ years	Baseline	3 years	Harrell's C: 0.82 CC = good calibration
Kauppi 2018^45^	Cox proportional hazards model	cross‐validation	Stable MCI versus Converter MCI	NI	Baseline	3 years	AUC = 0.84 ACC = 78.9%
Khajehpiri 2022^46^	Xgboost proportional hazard	10‐Fold cross‐validation	Stable MCI versus Converter MCI	Converter MCI: 30.2 (24.6) months Stable MCI: 47.8 (33.4) mean survival time	Baseline	During follow‐up	Harrell's C = 84.5%
Korolev 2016^47^	Probabilistic multiple kernel learning (pMKL) classification	10‐Fold cross‐validation	Stable MCI versus Converter MCI	NI	Baseline	3 years	AUC = 0.87 CCC = 0.95
Lee 2014^48^	Cox regression analysis	Bootstrapping	Stable MCI versus Converter MCI	Converter MCI: 2.9 years Stable MCI: 71 cases < 3 years; 132 cases ≥ 3 years	Baseline	3 years	Harrel's C = 0.71 CC = good calibration
Lee 2019^49^	Recurrent neural network	5‐Fold cross‐validation	Stable MCI versus Converter MCI	NI	Baseline^c^	2 years	ACC = 0.76
Luk 2018^50^	Binary logistic regression model	Random split in training and test set	Stable MCI versus Converter MCI	NI	Baseline	3 years	ACC = 76.2%
Mattila 2012^51^	Disease state index	10‐Fold cross‐validation	Stable MCI versus Converter MCI and indecisive group	NI	Baseline	2 years	^h^
Mubeen 2017^52^	Random Forest	OOB (out‐of‐bag) estimation and 10‐fold cross‐validation	Stable MCI versus Converter MCI	Converter MCI: 2.5 (1.8) years Stable MCI: 5.7 (2.3) years	Baseline	3 years	AUC = 0.82 ACC = 71.7%
					Longitudinal: 6 month	2.5 years	AUC = 0.87 ACC = 80.2%
Munoz‐Ruiz 2014^53^	Disease state index	Leave‐one‐out cross‐validation	Stable MCI versus Converter MCI	NI	Baseline	2.5 years^e^	ACC = 0.70
Pang 2023^54^	Random forest	5‐Fold cross‐validation	Stable MCI versus Converter MCI	NI	Baseline	2 years	AUC = 0.85 ACC = 85.3%
Park 2022^55^	LightGBM	Leave‐one‐out cross‐validation	Stable MCI versus Converter MCI	NI	Baseline	2 years	AUC = 0.79 ACC = 71.1%
Peng 2023^56^	Integrated machine learning: SVM, naïve Bayes, RF, KNN	10‐Fold cross‐validation	Stable MCI versus Converter MCI	NI	Baseline	8 years	AUC = 0.87
Platero 2020^57^	Linear mixed effects model	10‐Fold cross‐validation	Stable MCI versus Converter MCI	NI	Baseline	3 years	AUC = 0.86 ACC = 77.7%
					Longitudinal	2 years	AUC = 0.91 ACC = 81.6%
Platero 2021^58^	Linear mixed effects model	10‐Fold cross‐validation	Stable MCI versus Converter MCI	NI	Baseline	3 years	AUC = 0.86 ACC = 77.3%
					Longitudinal	2 years	AUC = 0.89 ACC = 80.9%
Runtti 2014^59^	Disease state index	10‐Fold cross‐validation	Stable MCI versus Converter MCI	NI	Longitudinal	3 years	AUC = 82.3% ACC = 76.9%
Shu 2021^60^	Support vector machine	Random split in training and test set	Stable MCI versus Converter MCI	NI	Baseline	3 years	AUC = 0.80 CC = good calibration
Tabatabaei‐Jafari 2018^61^	Discriminant analysis	10‐Fold cross‐validation	Stable + reverter^g^ MCI versus Converter MCI	NI	Baseline	5 years	AUC = 0.81 ACC = 74.6
Tam 2019^62^	Linear support vector machine	10‐Fold cross‐validation	Stable MCI versus Converter MCI	NI	Baseline	3 years	ACC = 85,1%^f^
Tang 2021^63^	Cox proportional hazards model	Random split in training and test set	Stable MCI versus Converter MCI	NI	Baseline	5 years	Harrel's C = 0.91 CC = good calibration
Varatharajah 2019^64^	Support vector machine – linear	5‐Fold cross‐validation	Stable MCI versus Converter MCI	NI	Baseline	3 years	AUC = 0.93 ACC = 0.81
Wang 2016^65^	Partial least squares analysis	Leave‐one‐out cross‐validation	Stable MCI versus Converter MCI	NI	Baseline	3 years	ACC = 86.1%
Wang 2023^66^	Bayesian Cox regression model	10‐Fold cross‐validation	Stable MCI versus Converter MCI	NI	Baseline	3 years	Harrel's C = 0.75 ICI = 0.06
Willette 2014^67^	Discriminant classification analysis	Leave‐10‐out cross‐validation	Stable MCI versus Converter MCI	NI	Baseline	2 years	AUC = 0.90 ACC = 83.3%
Wu 2023^68^	Logistic regression	5‐Fold cross‐validation	Stable MCI versus Converter MCI	NI	Baseline	3 years	AUC = 0.91 ACC = 83% CC = good calibration
Xu 2016^69^	Weighted multi‐modality sparse representation‐based classification	10‐Fold cross‐validation	Stable MCI versus Converter MCI	NI	Baseline	3 years	ACC = 82.5%
Yang 2012^70^	Linear support vector machine	Training in NC, MCI, and AD for classification. Leave‐one‐out cross‐validation Testing in MCI for prediction.	Stable MCI versus Converter MCI	NI	Baseline	2 years	ACC = 66.7%
Ye 2012^71^	Sparse logistic regression model	Leave‐one‐out cross‐validation	Stable MCI versus Converter MCI	NI	Baseline	4 years	AUC = 0.86
Zandifar 2020^72^	Naïve Bayes classifier	Leave‐one‐out cross‐validation	Stable MCI versus Converter MCI	NI	Baseline	Different time points	ACC = 81.3%
**Model development and validation studies (development + external validation)**							
Cui 2011^73^	Support Vector machine	Training data CN/AD; testing data MCI 10‐fold cross‐validation	Stable MCI versus Converter MCI	NI	Baseline	2 years	AUC = 0.80 ACC = 67.1%
Dukart 2015^74^	Naïve Bayes classification	Training data CN/AD; testing data MCI	Stable MCI versus Converter MCI	At least 2 year follow‐up	NI	2 years	ACC = 87%
Ezzati 2019^75^	Ensemble linear discriminant model	Training data CN/AD, 10‐fold cross‐validation; testing data MCI	Stable MCI versus Converter MCI	NI	Baseline	4 years	ACC = 77.0%
Hall 2015^76^	Disease State Index	10‐Fold cross‐validation and external validation	Stable MCI versus Converter MCI	ADNI: 2.9 years AddNeuroMed: 1 year DESCRIPA: 2.2 years Kuopio L‐MCI: 2.6 years	Baseline	During follow‐up	AUC = 0.74
Kruczyk 2012^77^	Monte Carlo feature selection (MCFS) and Rosetta for generating rule based models	10‐Fold cross‐validation and external validation	Stable MCI v Converter MCI	Malmo UH: stable MCI: 5.2 years	Baseline	4 years	AUC = 0.92
Ning 2018^78^	Neural network	L1 regularization Training and testing in CN/AD, validation in MCI	Stable MCI versus Converter MCI	NI	Baseline	2 years	AUC = 0.84
Tong 2017^79^	Random forest	10‐Fold cross‐validation Training data CN/AD; validation data MCI	Stable MCI versus Converter MCI	NI	Baseline	3 years	AUC = 0.87 ACC = 80.7%
van Maurik 2017^80^	Cox proportional hazards model	Internal validation (ADC cohort): 5‐fold cross‐validation External validation in ADNI cohort.	Stable MCI versus Converter MCI	2.4 (1.0) years	Baseline	3 years	Internal validation: Harrel's C = 0.70 External validation: Harrel's C = 0.73
van Maurik 2019^81^	Cox proportional hazards analysis	External validation on the ADC cohort (development in ADNI cohort)	Stable MCI versus Converter MCI	3^1^ years	Baseline	3 years	Internal validation: Harrel's C = 0.82 External validation: Harrel's C = 0.76
Van Maurik 2019^87^	Cox proportional hazards analysis	Split of the sample in train and test set (training in 3 of the database set and validation a separate database set)	Stable MCI versus Converter MCI	ADC = 2.4 (1.6) years ADNI‐2 = 2.6 (1.4) years ADNI = 3.3 (2.3) years EMIF‐AD = 2.2 (1.1) BioFINDER = 2.3 (1.3) years	Baseline	3 years	Harrel's C = 0.74 CC = good calibration
Westman 2012^82^	Orthogonal partial least squares	Training in AD and CTL, testing in MCI	Stable MCI versus Converter MCI	NI	Baseline	2 years	AUC = 0.61 ACC = 66.4%
Young 2013^83^	Gaussian process	Training in AD and healthy, testing in MCI	Stable MCI versus Converter MCI	NI	Baseline	3 years	AUC = 0.795 ACC = 74.1%
**Model validation studies (external validation)**							
Devenand 2012^84^	Validation QD study (Devenand 2008)	External validation	Stable MCI versus Converter MCI	NI	Baseline	3 years	AUC = 0.87 ACC = 77%
Liu 2013^85^	Validation of PredictND tool	External validation	Stable MCI versus Converter MCI	NI	Baseline	3 years	PredictND tool: ACC = 72% Clinician + PredictAD tool: ACC = 71%
Rhodius‐Meester 2016^86^	Validation of PredictND tool	External validation	Stable MCI versus Converter MCI	Median follow‐up 3 years (95% CI 2 ‐ 3 years)	Baseline	During follow‐up	AUC = 0.82
van Maurik 2019^87^	Validation of model proposed in Van Maurik 2017	Split of the sample in train and test set (Training in 3 of the database set and validation a separate database set)	Stable MCI versus Converter MCI	3^2^ years	Baseline	During follow‐up	Harrell's C = 0.74 CC = good calibration

Abbreviations: ACC, accuracy; AUC, area under the curve; CC, calibration curve; CCC, concordance correlation coefficient; Harrel's C, Harrel's c‐statistic; ICI, integrated calibration index; KNN, k‐nearest neighbours; MCI, mild cognitive impairment; NI, no information; RF, Random Forest; SC, subjective cognitive impairment; SVM, support vector machine.

^a^ In case of longitudinal data: prediction horizon is time from last longitudinal measurement until moment of outcome measurement.

^b^ in derivation set.

^c^ gradient boosting and extreme gradient boosting model showed comparable results.

^d^ also model developed based on longitudinal data. However, not clear at what the interval is between measurement longitudinal data and outcome measurement.

^e^ part of the data measured at baseline and part of the data measured as rate baseline to 6‐month measurement (prediction horizon = 2.5 years including the rate).

^f^ Only for people for which the prediction was labelled as high confidence.

^g^ Reverts to normal cognition after an initial diagnosis of MCI.

^h^ See Supplementary Table 4 for sensitivity and specificity values.

**TABLE 3:** Predictor sources per model

	Predictor sources							Outcome measures		
Source	Demographics	(Family) history	Genetics	Cognitive scores	MRI	PET	Fluid biomarkers	AUC^a^	ACC (%)	Cal^b^
**Model development studies**										
Adelson 2023^26^	✓	✓		✓				0.98	88.9	–
Ardekani 2016^27^	✓		✓	✓	✓			0.77	72.6	–
Bapat 2024^28^				✓	✓			0.86	79.9	–
Barnes 2014^29^				✓	✓			0.78	81.0	Good
Blazhenets 2020^30^			✓	✓		✓		0.87	–	–
Bouallègue 2017^31^				✓		✓		–	77	–
Cai 2023^32^	✓		✓	✓	✓			0.92	–	–
Cao 2023^33^	✓			✓		✓		0.79	73.3	–
Chang 2022^34^				✓	✓			0.75	68	–
Chun 2022^35^	✓		✓	✓				0.85	80.7	–
Devenand 2008^36^				✓	✓			0.95	89.7	–
Dobromsylin 2022^37^			✓		✓			0.80	–	–
El‐Sappagh 2021^38^				✓	✓	✓		0.87	87.1	–
Franciotti 2023^39^				✓			✓		89	–
Goel 2023^40^	✓		✓	✓	✓			0.90	89.4	–
Grassi 2019^41^	✓			✓				0.88	78.8	–
Hall 2015^42^			✓	✓	✓		✓	0.77	71	–
Hou 2023^43^	✓	✓	✓	✓	✓		✓	0.92	–	–
Jang 2017^44^	✓		✓					0.82	–	Good
Kauppi 2018^45^			✓	✓	✓			0.84	78.9	–
Khajehpiri 2022^46^	✓		✓	✓	✓			0.85	–	–
Korolev 2016^47^				✓	✓			0.87	–	0.95^c^
Lee 2014^48^	✓			✓				0.71	–	Good
Lee 2019^49^	✓		✓	✓	✓		✓	–	76	–
Luk 2018^50^	✓		✓	✓	✓				76.2	–
Mattila 2012^51^			✓	✓	✓		✓	–	–	–
Mubeen 2017^52^	✓		✓	✓	✓	✓		0.82	71.7	–
Munoz‐Ruiz 2014^53^			✓	✓	✓	✓	✓	–	70	–
Pang 2023^54^				✓	✓			0.85	85.3	–
Park 2022^55^	✓		✓	✓	✓			0.80	71.1	–
Peng 2023^56^				✓		✓		0.87	–	–
Platero 2020^57^				✓	✓			0.86	77.7	–
Platero 2021^58^	✓		✓	✓	✓			0.86	77.3	–
Runtti 2014^59^			✓	✓	✓		✓	0.82	76.9	–
Shu 2021^60^	✓		✓	✓	✓			0.80	–	Good
Tabatabaei‐Jafari 2018^61^				✓	✓			0.81	74.6	–
Tam 2019^62^	✓			✓	✓			–	85.1	–
Tang 2021^63^			✓	✓	✓		✓	0.91	–	Good
Varatharajah 2019 964)					✓	✓	✓	0.93	81	–
Wang 2016^65^					✓	✓		–	86.1	–
Wang 2023^66^	✓	✓		✓	✓	✓	✓	0.75	–	0.06^d^
Willette 2014^67^	✓			✓	✓		✓	0.90	83.3	–
Wu 2023^68^				✓	✓			0.91	83	Good
Xu 2016^69^					✓	✓		–	82.5	–
Yang 2012^70^					✓		✓	–	66.7	–
Ye 2012^71^			✓	✓	✓			0.86	–	–
Zandifar 2020^72^	✓			✓	✓	✓		–	81.3	–
**Model development and validation studies**										
Cui 2011^73^				✓	✓		✓	0.80	67.1	–
Dukart 2015^74^				✓	✓	✓		–	87	–
Ezzati 2019^75^	✓		✓		✓			–	77	–
Hall 2015^76^	✓		✓	✓	✓		✓	0.74	–	–
Kruczyk 2012^77^	✓		✓	✓			✓	0.92	–	–
Ning 2018^78^			✓		✓			0.84	–	–
Tong 2017^79^	✓			✓	✓			0.87	80.7	–
van Maurik 2017^80^	✓			✓	✓		✓	0.73	–	–
van Maurik 2019^81^	✓		✓	✓	✓	✓		0.76	–	–
Van Maurik 2019^87^	✓				✓		✓	0.74	–	Good
Westman 2012^82^					✓		✓	0.61	66.4	–
Young 2013^83^			✓		✓	✓		0.80	74.1	–
**Model validation studies**										
Devenand 2012^84^	✓			✓	✓			0.87	77	–
Liu 2013^85^	✓			✓	✓		✓	–	72	–
Rhodius‐Meester 2016^86^	✓			✓	✓		✓	0.82	–	–
van Maurik 2019^87^				✓			✓	0.74	–	Good
**Total**	31	3	28	53	52	15	21			

Abbreviations: ACC, accuracy; AUC, area under the curve; Cal, calibration.

^a^ AUC or Harrel's C;.

^b^ Calibration (assessed using a calibration curve).

^c^ Concordance correlation coefficient.

^d^ Integrated calibration index.

### 3.4 Quality assessment

**FIGURE 2:**
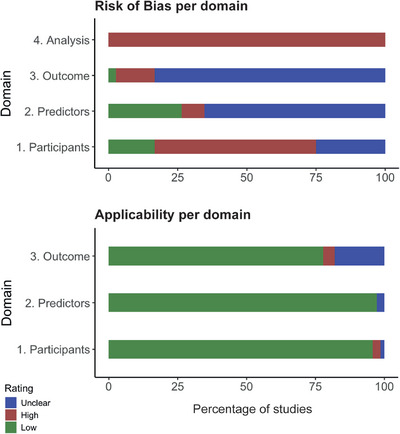
Risk of bias and applicability as assessed with the PROBAST tool.

The quality assessment using PROBAST is shown in Figure 2. Overall, most of the studies obtained an unclear or high risk of bias. Table S5 shows the assessment for the individual studies per domain.

#### 3.4.1 Risk of bias

Domain 1 of PROBAST covers potential concerns for bias related to sources of data and selection of participants. A high concern for risk of bias on domain 1 was frequently identified in the included studies for one of the following reasons: only cases with complete data were extracted from a database and included in the study for model development or validation; only cases for which data passed quality control were included for model development or validation; only cases with complete follow‐up or at least one follow‐up visit were included for model development or validation. Additionally, in many studies the methodology employed to address participants who were lost to follow‐up was unclear, and often specific inclusion and exclusion criteria to be included in the study were not provided. Domain 2 of PROBAST covers potential sources of bias related to the definition and measurement of predictors. In the studies that were included in this review, information on who performed the predictor assessments, how the predictors were assessed, and whether a standardized protocol was used for the measurements, was often lacking. Most of the studies, however, did refer to the standardized ADNI protocols which resulted in a positive valuation on the regarding sub‐questions. Domain 3 of PROBAST covers potential concerns of bias related to the definition and determination of the outcome. In many studies information was lacking on who performed the outcome measurement, the method of outcome determination and if a standardized protocol was used. Many studies again referred to the standardized ADNI protocols for outcome determination and received a positive score on the sub‐questions in domain 3 that were covered by following the ADNI protocols. Additionally, it is frequently unclear whether the outcome was determined without knowledge of the predictor information in the studies. Regarding predictors in the model, MMSE and/or CDR are often both included as a predictor in the model and used as a criterion to assess the outcome (part of the NINCDS‐ADRDA criteria for probable AD). Domain 4 of PROBAST examines if key statistical considerations were correctly addressed. A high overall risk for bias was identified in domain 4 for all studies. This was frequently due to the fact that studies did not include a sufficient number of participants with the outcome relative to the number of candidate predictors. PROBAST employs the criterion that the ratio of participants with the outcome relative to number of candidate predictor parameters should be ≥20,^20^ a number that most studies fall short of achieving. Often no information was provided on missing data and how this was handled. Additionally, often no information was provided on complexities in the data and how this was handled. Most studies did not report a calibration measure to express model performance.

#### 3.4.2 Applicability

The applicability of the included studies in addressing the research question of this systematic review was generally assessed as having a low risk for concern.

## 4 DISCUSSION

This systematic review comprehensively examines multivariable prediction models designed to estimate the risk of conversion from MCI to dementia. We included 62 studies that developed and/or validated a prediction model for this purpose. The studies in this review are included after a thorough selection process, in which single‐source prediction models were not considered. The reported performance measures of the models in this study suggest that the models seem reasonably effective at distinguishing between converter and stable MCI cases in the mid‐near future. However, external validation and methodological rigor, as assessed with the PROBAST tool, are often lacking.

Most prediction models in this review were based on machine learning methods (41 out of 62). In recent years, machine learning methods that enable a personalized health care approach through improved diagnosis and prediction have grown considerably.^88^ This review, however, does not show a difference in predictive performance of models based on machine learning compared to other model types to predict conversion to dementia among individuals with MCI. Wang et al. compared the predictive performance of various machine learning models to Cox regression models for predicting dementia in people with MCI. They showed negligible differences in the discrimination and calibration of Cox regression and machine learning models for this purpose.^89^ The number of predictors that are included in the models in this review ranged from 2 to 60. Including more predictors in a model could capture more comprehensive information of multifactor diseases like dementia and could therewith result in an improved predictive performance of a model. However, including a large number of predictors, especially in relation to the number of observations, can lead to overfitting.^22^ In addition, models with a larger number of predictors can become complex, making them harder to understand, interpret, and implement in clinical practice. In the studies that are included in this review, no clear trend in the type and number of predictors, related to the predictive performance could be observed. The number of predictors included in a model should find a balance between complexity and predictive performance. Moreover, including extra predictors may lead to an increased demand for resources, that is, costs and time. The effectiveness of a model depends not only on predictive performance, but also on other factors, including the quality of the data, the relevance of the selected features to the outcome, the model's ability to generalize to new, unseen data, and the clinical applicability.

The models included in this review mainly rely on the most established and clinically validated biomarkers for early detection of AD.^90^ Predictors derived from MRI, which is one of the most frequently used imaging modalities, are included in 52 of 62 the models. MRI is widely available and non‐invasive. However, MRI is considered to be both costly and time consuming, while structural changes in MRI images appear only late in the disease trajectory. PET, which was included in 15 of the 62 models, is an even more expensive and less available imaging technique compared to MRI. Previous cost‐effectiveness studies of individual sources for dementia risk estimation showed that PET is not cost‐effective when used in MCI to predict dementia.^91^ Fluid biomarkers, derived from blood or CSF, were used to obtain neurodegeneration specific protein markers in 18 of the 62 models. Although being invasive, lumbar puncture is a well‐known and reasonably well‐tolerated procedure with minimal side effects.^92^ A wide variety of cognitive scores was also frequently included as predictor in the models (53 of the 62 models). These tests are all relatively easy to administer, widely available, are less costly, and there is some good evidence that they can predict progression from MCI to dementia.^93^ Many different combinations of predictor sources were used in the prediction models. However, none of the combinations seems to outweigh the others in terms of predictive performance as presented in the papers.

Almost all studies used the ADNI database for developing a model. The goal of the ADNI database is to validate the use of biomarkers, including MRI and PET imaging, as well as other biological markers, to improve early detection and monitoring of AD. The focus lies on identifying pre‐dementia stages, enhancing intervention strategies at their most effective points, and promoting global research through an open‐access data policy.^94^ Since this dataset consists of a homogeneous sample of participants (87% white, 85% higher educated), this might lead to a limited generalizability of the models to different populations.^95^ Furthermore, the ADNI dataset lacks comorbidity heterogeneity, that is, subjects with known comorbidities for dementia were actively excluded. In real‐life cases, most individuals with MCI and dementia have varied comorbidities, as dementia is a known consequence of another primary disease in 40% of cases.^24^ ADNI and similarly the other cohorts, such as the ADC, DESCRIPA, and CREDOS, originate from specialized settings. Annual conversion rate in specialist settings is typically higher than in community based samples.^96^ Prediction models trained on this data may reflect the characteristics and disease progression of individuals who are more likely to convert from MCI to dementia due to being selected from clinical settings with rigorous diagnostic assessments and higher‐risk profiles. Consequently, this bias potentially affects the external validity of the models in community‐based settings.

Although the exact number of events per variable needed remains a topic of debate, the number of candidate predictors per case is often far below the suggested 20 events per variable that is used as a criterion on the PROBAST tool.^22^, ^97^, ^98^ In addition, the cohorts included in the different studies are often small, both leading to increased chances of bias and overfitting the models to a specific population. In addition, there is a general lack of external validation. The majority of the studies only performed internal validation (*n* = 47, 76%), whereas external validation is a crucial step toward defining the applicability of a model for its use in clinical practice.^22^ Considering the use of a model in clinical practice, most studies took the approach of evaluation the prognostic value in isolation, whereas this does not reflect clinical practice where a specialist will have additional patient information. Bruun et al., however, did study the use of a clinical AD prediction model in practice as a decision support tool for clinicians. Over a mean follow‐up period of 1.7 years, they showed that the overall prognostic accuracy was unaffected.^99^ Wang et al. tried to implement expert knowledge during model development. The predictive accuracy of models with or without expert knowledge did not differ significantly.^66^ All studies in this review were found to have a high risk for bias according to the assessment conducted with the PROBAST tool. Reporting on predictors and outcomes often was poor leading to an unclear risk for bias, whereas selection of participants and the model analysis often led to a high risk for bias. The high risk of bias prohibits drawing strong conclusions of the predictive ability and applicability of the models in this review.^100^ Although the PROBAST tool assesses the risk of bias and applicability of all sorts of prediction model studies, developed using both traditional statistical methods and modern machine learning techniques, the emergence of AI and machine learning has introduced specific nuances and challenges that PROBAST may not fully address. Consequently, a PROBAST‐AI tool is currently being developed to help critically appraise machine learning‐based prediction model studies.^101^ Additionally, the TRIPOD‐AI statement has recently been published to guide the reporting of prediction model studies, regardless of whether regression modelling or machine learning methods are used.^102^ In this systematic review, only two of the included studies mention a reporting guideline that was followed for conducting and reporting on the prediction model study.^66^, ^87^ In both papers, the TRIPOD guideline,^103^ which precedes the TRIPOD+AI statement was used. Improved adherence to the TRIPOD+AI statement, or other prediction model reporting guidelines, could help to consider all critical items during model development and subsequent reporting. It therefore has the potential to improve the quality and reporting of dementia risk prediction model development and validation studies.

### 4.1 Strengths and limitations of this systematic review

A strength of this review is that we used a broad search strategy, which resulted in many prediction models being retrieved. Prediction research is often difficult to identify.^104^ In our review, no additional papers were found through citation screening, suggesting that the relevant papers were found through the broad literature search. By restricting our search strategy to the databases PubMed and Embase, we kept a focused approach in searching for relevant literature. Together, PubMed and Embase provide the most comprehensive, specialized coverage in biomedical and clinical research.^105^, ^106^ Additionally, as previous reviews on dementia prediction models did not include an extensive risk of bias assessment,^14^, ^107^ this review provides additional important information on these aspects by using the PROBAST tool. This information could be used to improve the quality and reporting of future models, and therewith the translation of such models toward the clinic. A limitation of this systematic review is that we included studies with full‐text available in English, which could lead to language bias. However, during the full‐text screening phase, only one of all eligible studies appeared to be in a language other than English,^108^ so we do not believe that inclusion of this paper would have led to different results or findings.

Evidence indicates that a significant amount of dementia cases arise from modifiable risk factors (e.g., hypertension).^109^ It is presumed that early intervention in these MCI cases could result in prevention or delay of conversion to dementia.^109^ With the knowledge that up to 40% of people with MCI will convert to dementia within 5 years, it is believed to be important to identify those at risk for conversion.^96^ Identification of those at risk could help to target (future) therapies to those that will most likely benefit from it. Intervention early in the disease trajectory is thought to be imperative in delaying the progression of the disease,^4^, ^5^, ^6^ resulting in better health outcomes, a reduced demand for health care and additionally reduced overall health care expenditures.^110^ With today's standard clinical diagnostic procedures, no such efficient discrimination of MCI cases is possible. To be of added clinical value, a prediction model should be accurate, reliable, reproducible, cost‐effective, and easy to implement in large populations. Moreover, implementing such a prediction model in clinical practice should have a demonstrable positive effect on individuals and the health care system.

This systematic review shows that many prediction models for MCI to dementia conversion exist, but there is still work to do to warrant implementation. To define the generalizability and applicability of prediction models for use in clinical practice, crucial steps to be taken are external validation and improving the diversity of participants in datasets. Additionally, model evaluation should be performed under the circumstances that resemble the use in clinical practice (with prior clinical knowledge, but without knowing model predictors), and the reporting of prediction model studies should be improved. Moreover, focus should be on inclusion of predictors that are likely to be used in the future, easy to use and upscale, and cost‐effective. The limited generalizability and high risk of bias in most studies in this systematic review, prohibits strong conclusions about predictive performance and applicability of MCI to dementia prediction models. In summary, though many models have been developed, there still is no highly valid prediction model available for MCI to dementia conversion risk.

## CONFLICT OF INTEREST STATEMENT

The authors declare no conflicts of interest. Author disclosures are available in the supporting information.

## Supporting information



Supporting Information

Supporting Information

Supporting Information

Supporting Information

Supporting Information

Supporting Information

Supporting Information

Supporting Information

Supporting Information

Supporting Information

Supporting Information
