# Supplementary material for: Limited generalizability and high risk of bias in multivariable models predicting conversion risk from mild cognitive impairment to dementia: A systematic review
Source: Alzheimers Dement. 2025 Apr 6;21(4):e70069. doi: 10.1002/alz.70069 (PMC11972987; doi:10.1002/alz.70069)
Supplement: Supplementary file 2 — Supporting Information [file ALZ-21-e70069-s005.docx]

| **Supplementary figure 1A.** Predictors per source: demographics, (family) history, genetics. | | | | | | | | | | | | | | | | | | | | | | | | | | | | | | | | | |
| --- | --- | --- | --- | --- | --- | --- | --- | --- | --- | --- | --- | --- | --- | --- | --- | --- | --- | --- | --- | --- | --- | --- | --- | --- | --- | --- | --- | --- | --- | --- | --- | --- | --- |
|  | *Age* | *Sex* | *Marital status* | *Education* |  | *Diagnosis at baseline* | *Subtype of MCI* | *Visual or verbal MCI* | *Single or multiple domain MCI* | *Neuropsychiatric symptoms* | *Course of decline* | *Functional independence* | *Comorbidities* | *Smoking* | *Judgement and problem solving* | *Memory* | *Orientation* | *Independence* | *Depression* | *Psychosis* | *Medication (contributing to impairment)* | *Visuospatial function* | *Hyposomnia/insomnia* | *Vitamin B12 deficiency* | *Family medical history* |  | *APOE2* | *APOE4* | *APOE allele 1 and 2 (either ε2, ε3, or ε4)* | *​ SNPs* | *PHS* | *Complement receptor 1* |  |
| **Source** | **Demographics** | | | | |  | **(Family) history** | | | | | | | | | | | | | | | | | | | |  | **Genetics** | | | | | |
| *Adelson 2023 (26)* | 🗸 |  | 🗸 |  |  |  |  |  |  |  |  |  | 🗸 |  |  |  |  |  |  |  |  |  |  |  | 🗸 |  |  |  |  |  |  |  |  |
| *Ardekani 2016 (27)* | 🗸 | 🗸 |  | 🗸 |  |  |  |  |  |  |  |  |  |  |  |  |  |  |  |  |  |  |  |  |  |  |  | 🗸 |  |  |  |  |  |
| *Bapat 2024 (28)* |  |  |  |  |  |  |  |  |  |  |  |  |  |  |  |  |  |  |  |  |  |  |  |  |  |  |  | 🗸 |  |  |  |  |  |
| *Blazhenets 2020 (30)* |  |  |  |  |  |  |  |  |  |  |  |  |  |  |  |  |  |  |  |  |  |  |  |  |  |  |  | 🗸 |  |  |  |  |  |
| *Cai 2023 (32)* | 🗸 | 🗸 |  | 🗸 |  | 🗸 |  |  |  |  |  |  |  |  |  |  |  |  |  |  |  |  |  |  |  |  |  | 🗸 |  |  |  |  |  |
| *Cao 2023 (33)* | 🗸 |  |  |  |  |  |  |  |  |  |  |  |  |  |  |  |  |  |  |  |  |  |  |  |  |  |  |  |  |  |  |  |  |
| *Chun 2022 (35)* |  |  |  |  |  |  |  |  |  |  |  |  |  |  |  |  |  |  |  |  |  |  |  |  |  |  | 🗸 | 🗸 |  |  |  |  |  |
| *Devanand 2012 (84)* | 🗸 |  |  |  |  |  |  |  |  |  |  |  |  |  |  |  |  |  |  |  |  |  |  |  |  |  |  |  |  |  |  |  |  |
| *Dobromsylin 2022 (37)* |  |  |  |  |  |  |  |  |  |  |  |  |  |  |  |  |  |  |  |  |  |  |  |  |  |  |  | 🗸 |  |  |  |  |  |
| *Ezzati 2019 (75)* | 🗸 | 🗸 |  | 🗸 |  |  |  |  |  |  |  |  |  |  |  |  |  |  |  |  |  |  |  |  |  |  |  | 🗸 |  |  |  |  |  |
| *Franciotti 2023 (39)* |  |  |  |  |  |  |  |  |  |  |  |  |  |  |  |  |  |  |  |  |  |  |  |  |  |  |  | 🗸 |  |  |  |  |  |
| *Goel 2023 (40)* | 🗸 | 🗸 |  |  |  |  |  |  |  |  |  |  |  |  |  |  |  |  |  |  |  |  |  |  |  |  |  | 🗸 |  |  |  |  |  |
| *Grassi 2019 (41)* | 🗸 | 🗸 | 🗸 | 🗸 |  |  | 🗸 |  |  |  |  |  |  |  |  |  |  |  |  |  |  |  |  |  |  |  |  |  |  |  |  |  |  |
| *Hall 2015a (42)* |  |  |  |  |  |  |  |  |  |  |  |  |  |  |  |  |  |  |  |  |  |  |  |  |  |  |  | 🗸 |  |  |  |  |  |
| *Hall 2015b (76)* |  |  |  |  |  |  |  |  |  |  |  |  |  |  |  |  |  |  |  |  |  |  |  |  |  |  |  | 🗸 |  |  |  |  |  |
| *Hou 2023 (43)* |  |  |  |  |  |  |  |  |  |  |  |  |  |  |  |  |  |  | 🗸 |  |  |  |  |  |  |  |  |  |  |  | 🗸 |  |  |
| *Jang 2017 (44)* | 🗸 |  |  |  |  |  |  | 🗸 | 🗸 |  |  |  |  |  |  |  |  |  |  |  |  |  |  |  |  |  |  | 🗸 |  |  |  |  |  |
| *Kauppi 2018 (45)* |  |  |  |  |  |  |  |  |  |  |  |  |  |  |  |  |  |  |  |  |  |  |  |  |  |  |  |  |  |  | 🗸 |  |  |
| *Khajephiri 2022 (46)* | 🗸 | 🗸 |  | 🗸 |  |  |  |  |  |  |  |  |  |  |  |  |  |  |  |  |  |  |  |  |  |  |  | 🗸 |  |  |  |  |  |
| *Kruczyk 2012 (77)* | 🗸 | 🗸 |  |  |  |  |  |  |  |  |  |  |  |  |  |  |  |  |  |  |  |  |  |  |  |  |  |  |  |  |  |  |  |
| *Liu 2013 (85)* |  |  |  |  |  |  |  |  |  |  |  |  |  |  |  |  |  |  |  |  |  |  |  |  |  |  |  | 🗸 |  |  |  |  |  |
| *Lee 2014 (48)* |  | 🗸 |  |  |  |  |  |  |  | 🗸 |  | 🗸 |  |  |  |  |  |  |  |  |  |  |  |  |  |  |  |  |  |  |  |  |  |
| *Lee 2019 (49)* |  |  |  |  | ^*^ |  |  |  |  |  |  |  |  |  |  |  |  |  |  |  |  |  |  |  |  |  |  |  |  |  |  |  |  |
| *Luk 2018 (50)* |  | 🗸 |  |  |  |  |  |  |  |  |  |  |  |  |  |  |  |  |  |  |  |  |  |  |  |  |  | 🗸 |  |  |  |  |  |
| *Mattila 2012 (51)* |  |  |  |  |  |  |  |  |  |  |  |  |  |  |  |  |  |  |  |  |  |  |  |  |  |  |  | 🗸 |  |  |  |  |  |
| *Mubeen 2017 (52)* | 🗸 | 🗸 |  | 🗸 |  |  |  |  |  |  |  |  |  |  |  |  |  |  |  |  |  |  |  |  |  |  |  | 🗸 |  |  |  |  |  |
| *Ning 2018 (78)* |  |  |  |  |  |  |  |  |  |  |  |  |  |  |  |  |  |  |  |  |  |  |  |  |  |  |  | 🗸 |  | 🗸^**^ |  |  |  |
| *Pang 2023 (54)* |  |  |  |  |  |  |  |  |  |  | 🗸 |  |  | 🗸 | 🗸 | 🗸 | 🗸 | 🗸 | 🗸 |  | 🗸 | 🗸 | 🗸 |  |  |  |  | 🗸 |  |  |  |  |  |
| *Park 2022 (55)* | 🗸 | 🗸 |  | 🗸 |  |  |  |  |  |  |  |  |  |  |  |  |  |  |  |  |  |  |  |  |  |  |  | 🗸 |  |  |  |  |  |
| *Platero 2021 (58)* | 🗸 | 🗸 |  | 🗸 |  |  |  |  |  |  |  |  |  |  |  |  |  |  |  |  |  |  |  |  |  |  |  |  |  |  |  |  |  |
| *Rhodius-Meester 2016 (86)* | 🗸 |  |  | 🗸 |  |  |  |  |  |  |  |  |  |  |  |  |  |  |  |  |  |  |  |  |  |  |  | 🗸 |  |  |  |  |  |
| *Runtti 2014 (59)* |  |  |  |  |  |  |  |  |  |  |  |  |  |  |  |  |  |  |  |  |  |  |  |  |  |  |  |  | 🗸 |  |  |  |  |
| *Shu 2021 (60)* |  |  |  |  |  |  |  |  |  |  |  |  |  |  |  |  |  |  |  |  |  |  |  |  |  |  |  | 🗸 |  |  |  |  |  |
| *Tam 2019 962)* | 🗸 | 🗸 |  |  |  |  |  |  |  |  |  |  |  |  |  |  |  |  |  |  |  |  |  |  |  |  |  |  |  |  |  |  |  |
| *Tang 2021 (63)* |  |  |  |  |  |  |  |  |  |  |  |  |  |  |  |  |  |  |  |  |  |  |  |  |  |  |  | 🗸 |  |  |  |  |  |
| *Tong 2017 (79)* | 🗸 |  |  |  |  |  |  |  |  |  |  |  |  |  |  |  |  |  |  |  |  |  |  |  |  |  |  |  |  |  |  |  |  |
| *van Maurik 2017 (80)* | 🗸 |  |  |  |  |  |  |  |  |  |  |  |  |  |  |  |  |  |  |  |  |  |  |  |  |  |  |  |  |  |  |  |  |
| *van Maurik 2019a (81)* | 🗸 | 🗸 |  |  |  |  |  |  |  |  |  |  |  |  |  |  |  |  |  |  |  |  |  |  |  |  |  | 🗸 |  |  |  |  |  |
| *van Maurik 2019b (87)* | 🗸 |  |  |  |  |  |  |  |  |  |  |  |  |  |  |  |  |  |  |  |  |  |  |  |  |  |  |  |  |  |  |  |  |
| *Varatharajah 2019 (64)* | 🗸 |  |  |  |  |  |  |  |  |  |  |  |  |  |  |  |  |  |  |  |  |  |  |  |  |  |  |  |  |  |  | 🗸 |  |
| *Wang 2023 (66)* |  |  | 🗸 |  |  |  |  |  |  |  |  |  |  |  |  |  |  |  | 🗸 | 🗸 |  |  |  | 🗸 |  |  |  |  |  |  |  |  |  |
| *Willette 2014 (67)* | 🗸 | 🗸 |  | 🗸 |  |  |  |  |  |  |  |  |  |  |  |  |  |  |  |  |  |  |  |  |  |  |  |  |  |  |  |  |  |
| *Ye 2012 (71)* |  |  |  |  |  |  |  |  |  |  |  |  |  |  |  |  |  |  |  |  |  |  |  |  |  |  |  | 🗸 |  |  |  |  |  |
| *Young 2013 (83)* |  |  |  |  |  |  |  |  |  |  |  |  |  |  |  |  |  |  |  |  |  |  |  |  |  |  |  |  | 🗸 |  |  |  |  |
| *Zandifar 2020 (72)* | 🗸 | 🗸 |  | 🗸 |  |  |  |  |  |  |  |  |  |  |  |  |  |  |  |  |  |  |  |  |  |  |  |  |  |  |  |  |  |
| *Total* | 27 | 16 | 3 | 11 |  | 1 | 1 | 1 | 1 | 1 | 1 | 1 | 1 | 1 | 1 | 1 | 1 | 1 | 3 | 1 | 1 | 1 | 1 | 1 | 1 |  | 1 | 25 | 2 | 1 | 2 | 1 |  |

SNPs: Single Nucleotide Polymorphisms; PHS: Polygenic Hazard Score.

^*^4 demographic features in prediction model, not further specified; ^**^19 SNPs
