# Supplementary material for: Limited generalizability and high risk of bias in multivariable models predicting conversion risk from mild cognitive impairment to dementia: A systematic review
Source: Alzheimers Dement. 2025 Apr 6;21(4):e70069. doi: 10.1002/alz.70069 (PMC11972987; doi:10.1002/alz.70069)
Supplement: Supplementary file 3 — Supporting Information [file ALZ-21-e70069-s001.docx]

| **Supplementary figure 1B.** Predictors per source: cognitive tests. | | | | | | | | | | | | | | | | | | | | | | | | | | | | | | | | | | | | | | | | | | |
| --- | --- | --- | --- | --- | --- | --- | --- | --- | --- | --- | --- | --- | --- | --- | --- | --- | --- | --- | --- | --- | --- | --- | --- | --- | --- | --- | --- | --- | --- | --- | --- | --- | --- | --- | --- | --- | --- | --- | --- | --- | --- | --- |
|  | *NAB (neurophsychological assessment battery)* | *Learning* | *Memory* | *Fluency* | *Trail making test A and/or B* | *Modified Preclinical Alzheimer Cognitive Composite* | *Logical memory test* | *WAIS-R* | *Digit span forward and backward* | *Category fluency test* | *Boston naming test* | *Ideomotor apraxia* | *Calculation total score* | *Contrasting programm* | *go/no-go test* | *RCFT copy score* | *COWAT animal* | *AVLT* | *MMSE* | *GDS* | *CDR* | *CDR-SB* | *ADAS* | *ADAS-COG / ADAS-COG 11* | *ADAS-COG 13* | *Clock test score* | *FAQ score* | *Logical memory - delayed recall* | *SVLT* | *RCFT* | *AVLT* | *UPSIT* | *SRT immediate recall* | *RAVLT* | *NPI-Q* | *ADNI specific composite scoring* | *Logic memory subset of Wechslor memory scale revised* | *ADNI cognitive test scores* | *ANART* | *MoCA* | *Benson figure test* | *Modified Hachinski Ischemic score* |
| **Source** | **Cognitive tests** | | | | | | | | | | | | | | | | | | | | | | | | | | | | | | | | | | | | | | | | | |
| *Adelson 2023 (26)* | 🗸 |  |  |  |  |  |  |  |  |  |  |  |  |  |  |  |  |  | 🗸 | 🗸 | 🗸 |  |  | 🗸 |  |  | 🗸 |  |  |  |  |  |  | 🗸 | 🗸 | 🗸 |  |  |  |  |  | 🗸 |
| *Ardekani 2016 (27)* |  |  |  |  |  |  |  |  |  |  |  |  |  |  |  |  |  |  | 🗸 |  |  | 🗸 |  | 🗸 | 🗸 |  |  |  |  |  |  |  |  |  |  |  |  |  |  |  |  |  |
| *Bapat 2024 (28)* |  |  |  |  | 🗸 | 🗸^2^ | 🗸 |  |  |  |  |  |  |  |  |  |  |  | 🗸 |  |  | 🗸 |  | 🗸 | 🗸 |  | 🗸 |  |  |  |  |  |  | 🗸^3^ |  |  |  |  |  |  |  |  |
| *Barnes 2014 (29)* |  |  |  |  |  |  |  |  |  |  |  |  |  |  |  |  |  |  |  |  |  |  |  | 🗸 |  | 🗸 | 🗸 |  |  |  |  |  |  |  |  |  |  |  |  |  |  |  |
| *Blazhenets 2020 (30)* |  |  |  |  |  |  |  |  |  |  |  |  |  |  |  |  |  |  | 🗸 |  |  |  |  |  |  |  | 🗸 |  |  |  |  |  |  |  |  |  |  |  |  |  |  |  |
| *Bouallègue 2017 (31)* |  |  |  |  |  |  |  |  |  |  |  |  |  |  |  |  |  |  |  |  |  |  |  | 🗸 |  |  |  |  |  |  |  |  |  |  |  |  |  |  |  |  |  |  |
| *Cai 2023 (32)* |  |  |  |  |  |  |  |  |  |  |  |  |  |  |  |  |  |  | 🗸 |  |  | 🗸 |  |  | 🗸 |  | 🗸 |  |  |  |  |  |  |  |  |  |  |  |  |  |  |  |
| *Cao 2023 (33)* |  |  |  |  |  | 🗸^2^ |  |  |  |  |  |  |  |  |  |  |  |  | 🗸 |  |  | 🗸 |  |  |  |  |  | 🗸 |  |  |  |  |  |  |  |  |  |  |  |  |  |  |
| *Chang 2022 (34)* |  |  |  |  | 🗸^2^ |  |  |  | 🗸 | 🗸 | 🗸 |  |  |  |  |  |  |  |  |  |  |  |  | 🗸^3^ |  | 🗸 |  |  |  |  |  |  |  | 🗸 |  |  |  |  |  |  |  |  |
| *Chun 2022 (35)* |  |  |  |  |  |  |  |  |  |  | 🗸 | 🗸 | 🗸 | 🗸 | 🗸 | 🗸 | 🗸 |  | 🗸 |  |  | 🗸 |  |  |  |  |  |  | 🗸^2^ | 🗸^2^ |  |  |  |  |  |  |  |  |  |  |  |  |
| *Cui 2011 (73)* |  |  |  |  |  |  | 🗸 |  |  |  |  |  |  |  |  |  |  |  |  |  |  |  |  |  |  |  | 🗸 |  |  |  | 🗸^2^ |  |  |  |  |  |  |  |  |  |  |  |
| *Devenand 2008 (36)* |  |  |  |  |  |  |  |  |  |  |  |  |  |  |  |  |  |  |  |  |  |  |  |  |  |  | 🗸 |  |  |  |  | 🗸 | 🗸 |  |  |  |  |  |  |  |  |  |
| *Devanand 2012 (84)* |  |  |  |  |  |  |  |  |  |  |  |  |  |  |  |  |  |  | 🗸 |  |  |  |  |  |  |  | 🗸 |  |  |  | 🗸 |  | 🗸 |  |  |  |  |  |  |  |  |  |
| *Dukart 2015 (74)* |  |  |  |  |  |  |  |  |  |  |  |  |  |  |  |  |  |  | 🗸 | 🗸 |  |  |  | 🗸 |  |  | 🗸 |  |  |  |  |  |  | 🗸 |  |  |  |  |  |  |  |  |
| *El-Sappagh 2021 (38)* | 🗸 |  |  |  |  |  |  |  |  |  |  |  |  |  |  |  |  |  |  |  |  |  |  |  |  |  |  |  |  |  |  |  |  |  |  |  |  |  |  |  |  |  |
| *Franciotti 2023 (39)* |  |  |  |  |  |  |  |  |  |  | 🗸 |  |  |  |  |  |  |  |  |  |  | 🗸 |  | 🗸 | 🗸 | 🗸 | 🗸 |  |  |  |  |  |  |  |  |  |  |  |  |  |  |  |
| *Goel 2023 (40)* |  |  |  |  |  |  |  |  |  |  |  |  |  |  |  |  |  |  | 🗸 |  |  | 🗸 |  | 🗸 | 🗸 |  |  |  |  |  |  |  |  |  |  |  |  |  |  |  |  |  |
| *Grassi 2019 (41)* |  |  |  |  | 🗸 |  |  |  |  |  |  |  |  |  |  |  |  |  | 🗸 |  |  | 🗸 | 🗸 |  |  |  | 🗸 |  |  |  |  |  |  | 🗸^3^ |  |  | 🗸 |  |  |  |  |  |
| *Hall 2015a (42)* |  | 🗸 | 🗸 | 🗸 | 🗸^2^ |  |  |  |  |  |  |  |  |  |  |  |  |  | 🗸 |  |  |  |  |  |  |  |  |  |  |  |  |  |  |  |  |  |  |  |  |  |  |  |
| *Hall 2015b (76)* |  |  |  |  |  |  |  |  |  |  |  |  |  |  |  |  |  |  | 🗸 |  |  |  |  |  |  |  |  |  |  |  |  |  |  |  |  |  |  |  |  |  |  |  |
| *Hou 2023 (43)* |  |  |  |  |  |  | 🗸 |  |  |  |  |  |  |  |  |  |  |  | 🗸 |  |  |  |  | 🗸 |  |  | 🗸 |  |  |  |  |  |  | 🗸 |  |  |  |  |  |  |  |  |
| *Kauppi 2018 (45)* |  |  |  |  |  |  |  |  |  |  |  |  |  |  |  |  |  |  | 🗸 |  |  |  |  |  |  |  |  |  |  |  |  |  |  |  |  |  |  |  |  |  |  |  |
| *Khajephiri 2022 (46)* |  |  |  |  |  |  |  |  |  |  |  |  |  |  |  |  |  |  |  |  |  | 🗸 |  |  | 🗸 |  | 🗸 |  |  |  |  |  |  |  |  |  |  | 🗸^4^ |  |  |  |  |
| *Korolev 2016 (47)* |  |  |  |  |  |  |  |  |  |  |  |  |  |  |  |  |  |  |  |  |  |  |  | 🗸 |  |  | 🗸 |  |  |  |  |  |  | 🗸 |  |  |  |  |  |  |  |  |
| *Lee 2014 (48)* |  |  |  |  |  |  |  |  |  |  |  |  |  |  |  |  |  |  |  |  |  |  |  | 🗸^2^ |  | 🗸 |  |  |  |  |  |  |  |  |  |  |  |  |  |  |  |  |
| *Lee 2019 (49)* | ^*^ |  |  |  |  |  |  |  |  |  |  |  |  |  |  |  |  |  |  |  |  |  |  |  |  |  |  |  |  |  |  |  |  |  |  |  |  |  |  |  |  |  |
| *Liu 2013 (85)* |  |  |  |  | 🗸 |  | 🗸 | 🗸 | 🗸 | 🗸 | 🗸 |  |  |  |  |  |  | 🗸 |  |  |  |  |  | 🗸 |  | 🗸 |  |  |  |  | 🗸 |  |  |  |  |  |  |  | 🗸 |  |  |  |
| *Luk 2018 (50)* |  |  |  |  |  |  |  |  |  |  |  |  |  |  |  |  |  |  | 🗸 |  |  |  |  |  |  |  |  |  |  |  |  |  |  |  |  |  |  |  |  |  |  |  |
| *Mattila 2012 (51)* |  |  |  |  | 🗸 |  | 🗸 | 🗸 | 🗸 | 🗸 | 🗸 |  |  |  |  |  |  | 🗸 |  |  |  |  |  | 🗸 |  | 🗸 |  |  |  |  | 🗸 |  |  |  |  |  |  |  | 🗸 |  |  |  |
| *Mubeen 2017 (52)* |  |  |  |  |  |  |  |  |  |  |  |  |  |  |  |  |  |  | 🗸 |  |  | 🗸 |  |  | 🗸 |  | 🗸 |  |  |  |  |  |  |  |  |  |  |  |  |  |  |  |
| *Munoz-Ruiz 2014 (53)* |  |  |  |  | 🗸 |  | 🗸 | 🗸 | 🗸 | 🗸 | 🗸 |  |  |  |  |  |  | 🗸 | 🗸 |  |  |  | 🗸 |  |  | 🗸^2^ |  |  |  |  | 🗸 |  |  |  |  |  |  |  | 🗸 |  |  |  |
| *Pang 2023 (54)* | 🗸 |  |  |  | 🗸 |  |  |  |  |  |  |  |  |  |  |  |  |  |  |  |  |  |  |  |  |  |  |  |  |  |  |  |  |  |  |  |  |  |  |  | 🗸 |  |
| *Park 2022 (55)* |  |  |  |  |  |  |  |  |  |  |  |  |  |  |  |  |  |  | 🗸 |  |  | 🗸 |  | 🗸 |  |  |  |  |  |  |  |  |  |  |  |  |  |  |  |  |  |  |
| *Peng 2023 (56)* |  |  |  |  |  |  |  |  |  |  |  |  |  |  |  |  |  |  |  |  | 🗸 |  | 🗸 |  |  |  |  |  |  |  |  |  |  |  |  |  |  |  |  |  |  |  |
| *Platero 2020 (57)* |  |  |  |  |  |  |  |  |  |  |  |  |  |  |  |  |  |  |  |  |  |  | 🗸 |  | 🗸 |  | 🗸 |  |  |  |  |  |  | 🗸 |  |  |  |  |  |  |  |  |
| *Platero 2021 (58)* |  |  |  |  |  |  |  |  |  |  |  |  |  |  |  |  |  |  |  |  | 🗸 |  |  |  | 🗸 |  | 🗸 |  |  |  |  |  |  | 🗸 |  |  |  |  |  |  |  |  |
| *Rhodius-Meester 2016 (86)* |  |  |  |  | 🗸 |  | 🗸 | 🗸 | 🗸 | 🗸 | 🗸 |  |  |  |  |  |  | 🗸 |  |  |  |  |  | 🗸 |  | 🗸 |  |  |  |  | 🗸 |  |  |  |  |  |  |  | 🗸 |  |  |  |
| *Runtti 2014 (59)* |  |  |  |  | 🗸 |  | 🗸^2^ | 🗸 | 🗸 | 🗸 | 🗸 |  |  |  |  |  |  | 🗸 | 🗸 |  |  |  |  |  | 🗸 | 🗸^2^ |  |  |  |  | 🗸 |  |  |  |  |  |  |  | 🗸 |  |  |  |
| *Shu 2021 (60)* |  |  |  |  |  |  |  |  |  |  |  |  |  |  |  |  |  |  |  |  | 🗸 |  | 🗸 |  |  |  |  |  |  |  |  |  |  |  |  |  |  |  |  |  |  |  |
| *Tabatabaei-Jafari 2018 (61)* | |  |  |  |  |  |  |  |  |  |  |  |  |  |  |  |  |  | 🗸 |  |  |  |  |  |  |  |  |  |  |  |  |  |  |  |  |  |  |  |  |  |  |  |
| *Tam 2019 (62)* |  |  |  |  |  |  |  |  |  |  | 🗸 |  |  |  |  |  |  |  |  |  |  |  |  |  | 🗸 | 🗸 |  |  |  |  |  |  |  |  |  |  |  | 🗸 |  |  |  |  |
| *Tang 2021 (63)* |  |  |  |  |  |  |  |  |  |  |  |  |  |  |  |  |  | 🗸 | 🗸 |  | 🗸 |  |  | 🗸 | 🗸 |  | 🗸 |  |  |  |  |  |  | 🗸^4^ |  |  |  |  |  |  |  | 🗸 |
| *Tong 2017 (79)* |  |  |  |  |  |  |  |  |  |  |  |  |  |  |  |  |  |  | 🗸 |  |  | 🗸 |  | 🗸 |  |  | 🗸 |  |  |  |  |  |  | 🗸 |  |  |  |  |  |  |  |  |
| *van Maurik 2017 (80)* |  |  |  |  |  |  |  |  |  |  |  |  |  |  |  |  |  |  | 🗸 |  |  |  |  |  |  |  |  |  |  |  |  |  |  |  |  |  |  |  |  |  |  |  |
| *van Maurik 2019a (81)* |  |  |  |  |  |  |  |  |  |  |  |  |  |  |  |  |  |  | 🗸 |  |  |  |  |  |  |  |  |  |  |  |  |  |  |  |  |  |  |  |  |  |  |  |
| *van Maurik 2019b (87)* |  |  |  |  |  |  |  |  |  |  |  |  |  |  |  |  |  |  | 🗸 |  |  |  |  |  |  |  |  |  |  |  |  |  |  |  |  |  |  |  |  |  |  |  |
| *van Maurik 2019b – validation (87)* |  |  |  |  |  |  |  |  |  |  |  |  |  |  |  |  |  |  | 🗸 |  |  |  |  |  |  |  |  |  |  |  |  |  |  |  |  |  |  |  |  |  |  |  |
| *Wang 2023 (66)* |  |  |  |  |  |  |  |  |  |  |  |  |  |  |  |  |  |  | 🗸 |  |  |  |  |  |  |  |  |  |  |  |  |  |  |  |  |  |  |  |  |  |  |  |
| *Willette 2014 (67)* |  |  |  |  |  |  |  |  |  |  |  |  |  |  |  |  |  |  |  |  |  |  |  |  |  |  |  |  |  |  |  |  |  |  |  |  |  | 🗸^2^ |  |  |  |  |
| *Wu 2023 (68)* |  |  |  |  | 🗸 |  |  |  |  |  |  |  |  |  |  |  |  |  |  |  |  |  |  | 🗸 |  |  | 🗸 |  |  |  |  |  |  | 🗸 |  |  |  |  |  |  |  |  |
| *Ye 2012 (71)* |  |  |  |  | 🗸 |  |  |  |  |  |  |  |  |  |  |  |  |  |  |  |  |  |  | 🗸^4^ |  |  | 🗸 | 🗸 |  |  |  |  |  |  |  |  |  |  |  |  |  |  |
| *Zandifar 2020 (72)* |  |  |  |  |  |  |  |  |  |  |  |  |  |  |  |  |  |  | 🗸 |  |  | 🗸 |  | 🗸 |  |  |  |  |  |  |  |  |  | 🗸 |  |  |  |  |  |  |  |  |
| *Total* | 3 | 1 | 1 | 1 | 12 | 2 | 8 | 5 | 6 | 6 | 9 | 1 | 1 | 1 | 1 | 1 | 1 | 6 | 29 | 2 | 5 | 13 | 5 | 21 | 12 | 10 | 21 | 2 | 1 | 1 | 7 | 1 | 2 | 13 | 1 | 1 | 1 | 3 | 5 | 0 | 1 | 2 |

^*^2 cognitive performance features, not further specified.

Superscript numbers indicate the number of predictors extracted from cognitive test (e.g. subdomains from RAVLT).
