# Supplementary material for: Limited generalizability and high risk of bias in multivariable models predicting conversion risk from mild cognitive impairment to dementia: A systematic review
Source: Alzheimers Dement. 2025 Apr 6;21(4):e70069. doi: 10.1002/alz.70069 (PMC11972987; doi:10.1002/alz.70069)
Supplement: Supplementary file 4 — Supporting Information [file ALZ-21-e70069-s006.docx]

| **Supplementary figure 1C.** Predictors per source: MRI. | | | | | | | | | | | | | | | | | | | | | | | | | | | | | | | | | | | | | | | | | | | |
| --- | --- | --- | --- | --- | --- | --- | --- | --- | --- | --- | --- | --- | --- | --- | --- | --- | --- | --- | --- | --- | --- | --- | --- | --- | --- | --- | --- | --- | --- | --- | --- | --- | --- | --- | --- | --- | --- | --- | --- | --- | --- | --- | --- |
|  | *MRI - general* | *Whole brain volume* | *Whole brain texture* | *Brainstem volume* | *Grey matter volume* | *Intracranial volume* | *Cortical Atrophy* | *Subcortical Atrophy* | *Regional atrophy* | *Cerebral cortex* |  | *Cerebral white matter* | *Cerebellum cortex* | *Cerebellum white matter* | *Comprehensive visual rating scale* | *Hippocampus* | *Temporal lobe* | *Total brain parenchyma* | *Small vessel disease* | *Corpus callosum* | *Radiomics Signature* | *Cranial volume (ICV)* | *Thalamus* |  | *Ventral diencephalon* | *Pallidum* | *Entorhinal cortex* | *Temporal cortex* | *Transverse temporal cortex* | *Insular cortex* | *Parietal Lobe* | *Calcarine cortex* | *Parietal cortex* | *Parietal Lobe* | *Temporal lobe* | *Temporal gyrus* | *Temporal sulcus* | *Paracentral sulcus* | *Postcentral gyrus* | *Precentral gyrus* | *Frontal operculum* | *Orbital operculum* |  |
| **Source** | **MRI** | | | | | | | | | | | | | | | | | | | | | | | | | | | | | | | | | | | | | | | | | | |
| *Ardekani 2016 (27)* |  |  |  |  |  |  |  |  |  |  |  |  |  |  |  | 🗸^2^ |  |  |  |  |  |  |  |  |  |  |  |  |  |  |  |  |  |  |  |  |  |  |  |  |  |  |  |
| *Bapat 2024 (28)* | 🗸 |  |  |  |  |  |  |  |  |  |  |  |  |  |  |  |  |  |  |  |  |  |  |  |  |  |  |  |  |  |  |  |  |  |  |  |  |  |  |  |  |  |  |
| *Barnes 2014 (29)* |  |  |  |  |  |  |  |  |  |  |  |  |  |  |  | 🗸 |  |  |  |  |  |  |  |  |  |  |  | 🗸 |  |  |  |  |  |  |  |  |  |  |  |  |  |  |  |
| *Cai 2023 (32)* |  |  |  |  |  |  |  |  |  |  |  |  |  |  |  | 🗸^2^ |  |  |  |  |  |  |  |  |  |  | 🗸^4^ | 🗸^4^ |  |  |  |  |  |  |  |  |  |  |  |  |  |  |  |
| *Cui 2011 (73)* |  |  |  |  |  |  |  |  |  |  |  |  |  |  |  | 🗸^2^ |  |  |  |  |  |  |  |  |  |  | 🗸 |  |  |  |  |  | 🗸 |  |  | 🗸 |  |  |  |  |  |  |  |
| *Devenand 2008 (36)* |  |  |  |  |  |  |  |  |  |  |  |  |  |  |  | 🗸 |  |  |  |  |  |  |  |  |  |  | 🗸 |  |  |  |  |  |  |  |  | 🗸 |  |  |  |  |  |  |  |
| *Devenand 2012 (84)* |  |  |  |  |  |  |  |  |  |  |  |  |  |  |  | 🗸 |  |  |  |  |  |  |  |  |  |  | 🗸 |  |  |  |  |  |  |  |  |  |  |  |  |  |  |  |  |
| *Dobromsylin 2022 (37)* |  |  |  |  |  |  |  |  |  |  |  |  |  |  |  | 🗸 |  |  |  |  |  |  |  |  |  |  |  |  |  |  |  |  |  |  |  |  |  |  |  |  |  |  |  |
| *Dukart 2015 (74)* | 🗸 |  |  |  |  |  |  |  |  |  |  |  |  |  |  |  |  |  |  |  |  |  |  |  |  |  |  |  |  |  |  |  |  |  |  |  |  |  |  |  |  |  |  |
| *El-Sappagh 2021 (38)* | 🗸^2^ |  |  |  |  |  |  |  |  |  |  |  |  |  |  |  |  |  |  |  |  |  |  |  |  |  |  |  |  |  |  |  |  |  |  |  |  |  |  |  |  |  |  |
| *Ezzati 2019 (75)* | 🗸^47^ |  |  |  |  |  |  |  |  |  |  |  |  |  |  | 🗸 |  |  |  |  |  |  |  |  |  |  |  |  |  |  |  |  |  |  |  |  |  |  |  |  |  |  |  |
| *Goel 2023 (40)* |  |  |  |  |  |  |  |  |  |  |  |  |  |  |  |  |  |  |  | 🗸 |  |  |  |  |  |  |  |  |  |  |  |  |  |  |  |  |  |  |  |  |  |  |  |
| *Hall 2015a (42)* |  |  |  |  |  |  |  |  |  |  |  |  |  |  |  | 🗸^3^ |  |  |  |  |  |  |  |  |  |  |  |  |  |  |  |  |  |  | 🗸 |  |  |  |  |  |  |  |  |
| *Hall 2015b (76)* |  |  |  |  |  |  |  |  |  |  |  |  |  |  |  | 🗸 |  |  |  |  |  |  |  |  |  |  |  |  |  |  |  |  |  |  |  |  |  |  |  |  |  |  |  |
| *Hou 2023 (43)* |  | 🗸 |  |  |  |  |  |  |  |  |  |  |  |  |  | 🗸 |  |  |  |  |  |  |  |  |  |  |  |  |  |  |  |  |  |  |  |  |  |  |  |  |  |  |  |
| *Kauppi 2018 (45)* |  |  |  |  |  |  |  |  |  |  |  |  |  |  |  | 🗸 |  |  |  |  |  |  |  |  |  |  | 🗸 |  |  |  |  |  |  |  |  | 🗸^2^ | 🗸 |  |  |  |  |  |  |
| *Khajephiri 2022 (46)* |  |  |  |  |  |  |  |  |  |  |  |  |  |  |  | 🗸^2^ |  |  |  |  |  |  |  |  |  |  |  |  |  |  |  |  |  |  |  | 🗸 |  |  |  |  |  |  |  |
| *Korolev 2016 (47)* |  |  |  |  |  |  |  |  |  |  |  |  |  |  |  | 🗸 |  |  |  |  |  |  |  |  |  |  |  | 🗸 |  |  |  |  | 🗸 |  |  |  |  |  |  |  |  |  |  |
| *Lee 2019 (49)* | 🗸^3^ |  |  |  |  |  |  |  |  |  |  |  |  |  |  |  |  |  |  |  |  |  |  |  |  |  |  |  |  |  |  |  |  |  |  |  |  |  |  |  |  |  |  |
| *Liu 2013 (85)* |  |  |  |  |  |  |  |  |  |  |  |  |  |  |  | 🗸 |  |  |  |  |  |  |  |  |  |  |  |  |  |  |  |  |  |  |  |  |  |  |  |  |  |  |  |
| *Luk 2018 (50)* |  |  | 🗸 |  |  |  |  |  |  |  |  |  |  |  |  | 🗸 |  |  |  |  |  |  |  |  |  |  |  |  |  |  |  |  |  |  |  |  |  |  |  |  |  |  |  |
| *Mattila 2012 (51)* |  |  |  |  |  |  |  |  |  |  |  |  |  |  |  | 🗸 |  |  |  |  |  |  |  |  |  |  |  |  |  |  |  |  |  |  |  |  |  |  |  |  |  |  |  |
| *Mubeen 2017 (52)* |  |  |  |  |  |  |  |  |  |  |  |  |  |  |  | 🗸^4^ |  |  |  | 🗸^2^ |  |  |  |  |  |  |  |  |  |  |  |  |  |  |  |  |  |  |  |  |  |  |  |
| *Munoz-Ruiz 2014 (53)* |  |  |  |  |  |  |  |  | 🗸 |  |  |  |  |  |  | 🗸 | 🗸 |  |  |  |  |  |  |  |  |  |  |  |  |  |  |  |  |  |  |  |  |  |  |  |  |  |  |
| *Ning 2018 (78)* |  |  |  |  |  |  |  |  |  |  |  |  |  |  |  | 🗸^4^ |  |  |  |  |  |  |  |  |  |  | 🗸^2^ | 🗸^2^ |  |  | 🗸^4^ |  |  | 🗸 |  | 🗸^2^ | 🗸^2^ |  |  |  |  |  |  |
| *Pang 2023 (54)* |  |  |  |  |  |  |  |  |  |  |  |  |  |  |  | 🗸 |  |  |  |  |  |  |  |  |  |  |  |  |  |  |  |  |  |  |  |  |  |  |  |  |  |  |  |
| *Park 2022 (55)* |  |  |  |  |  |  | 🗸 | 🗸 |  |  |  |  |  |  | 🗸 | 🗸 |  |  | 🗸 |  |  |  |  |  |  |  |  |  |  |  |  |  |  |  |  |  |  |  |  |  |  |  |  |
| *Platero 2020 (57)* |  |  |  |  |  |  |  |  |  |  |  |  |  |  |  | 🗸 | 🗸 |  |  |  |  |  |  |  |  |  |  |  |  |  |  |  |  |  |  |  |  |  |  |  |  |  |  |
| *Platero 2021 (58)* |  |  |  |  |  |  |  |  |  |  |  |  |  |  |  | 🗸 | 🗸 |  |  |  |  |  |  |  |  |  |  |  |  |  |  |  |  |  |  |  |  |  |  |  |  |  |  |
| *Rhodius-Meester 2016 (86)* |  |  |  |  |  |  |  |  |  |  |  |  |  |  |  | 🗸 |  |  |  |  |  |  |  |  |  |  |  |  |  |  |  |  |  |  |  |  |  |  |  |  |  |  |  |
| *Runtti 2014 (59)* |  |  |  |  |  |  |  |  |  |  |  |  |  |  |  | 🗸^2^ |  | 🗸 |  |  |  |  |  |  |  |  | 🗸^2^ | 🗸^2^ |  |  |  |  |  |  | 🗸^2^ |  |  |  |  |  |  |  |  |
| *Shu 2021 (60)* |  |  |  |  |  |  |  |  |  |  |  |  |  |  |  |  |  |  |  |  | 🗸^10^ |  |  |  |  |  |  |  |  |  |  |  |  |  |  |  |  |  |  |  |  |  |  |
| *Tabatabaei-Jafari 2018 (61)* |  |  |  |  |  | 🗸 |  |  |  |  |  |  |  |  |  | 🗸 |  |  |  |  |  |  |  |  |  |  |  |  |  |  |  |  |  |  |  |  |  |  |  |  |  |  |  |
| *Tam 2019 (62)* |  |  |  |  | 🗸 | 🗸 |  |  |  |  |  |  |  |  |  |  |  |  |  |  |  |  |  |  |  |  |  |  |  |  |  |  |  |  |  |  |  |  |  |  |  |  |  |
| *Tang 2021 (63)* |  |  |  |  |  |  |  |  |  |  |  |  |  |  |  |  |  |  |  |  | 🗸^33^ |  |  |  |  |  |  |  |  |  |  |  |  |  |  |  |  |  |  |  |  |  |  |
| *Tong 2017 (79)* |  |  |  |  |  |  |  |  |  |  |  |  |  |  |  |  |  |  |  |  |  |  |  |  |  |  |  |  |  |  |  |  |  |  |  |  |  |  |  |  |  |  |  |
| *van Maurik 2017 (80)* |  | 🗸 |  |  |  |  |  |  |  |  |  |  |  |  |  |  |  |  |  |  |  |  |  |  |  |  |  |  |  |  |  |  |  |  |  |  |  |  |  |  |  |  |  |
| *van Maurik 2019a (81)* |  |  |  |  |  |  |  |  |  |  |  |  |  |  |  | 🗸 |  |  |  |  |  |  |  |  |  |  |  |  |  |  |  |  |  |  |  |  |  |  |  |  |  |  |  |
| *van Maurik 2019b (87)* |  |  |  |  |  |  |  |  |  |  |  |  |  |  |  | 🗸 |  |  |  |  |  |  |  |  |  |  |  |  |  |  |  |  |  |  |  |  |  |  |  |  |  |  |  |
| *Varatharajah 2019 (64)* |  |  |  |  |  |  |  |  |  |  |  |  |  |  |  | 🗸 |  |  |  |  |  |  |  |  |  |  |  |  |  | 🗸 |  |  | 🗸 |  |  |  |  |  |  |  |  |  |  |
| *Wang 2016 (65)* | 🗸 |  |  |  |  |  |  |  |  |  |  |  |  |  |  |  |  |  |  |  |  |  |  |  |  |  |  |  |  |  |  |  |  |  |  |  |  |  |  |  |  |  |  |
| *Wang 2023 (66)* |  |  |  |  |  |  |  |  |  |  |  |  |  |  |  | 🗸 |  |  |  |  |  |  |  |  |  |  |  |  |  |  |  |  |  |  |  |  |  |  |  |  |  |  |  |
| *Westman 2012 (82)* |  |  |  | 🗸 |  |  |  |  |  | 🗸^2^ |  | 🗸^2^ | 🗸^2^ | 🗸^2^ |  | 🗸^2^ |  |  |  | 🗸^5^ |  |  | 🗸^3^ |  | 🗸^2^ | 🗸^2^ | 🗸^2^ | 🗸^2^ | 🗸^2^ | 🗸^2^ | 🗸^2^ | 🗸^2^ | 🗸^2^ |  |  | 🗸^2^ | 🗸^2^ | 🗸^2^ | 🗸^2^ | 🗸^2^ | 🗸^2^ | 🗸^2^ |  |
| *Willette 2014 (67)* |  | 🗸^30^ |  |  |  |  |  |  |  |  |  |  |  |  |  |  |  |  |  |  |  |  |  |  |  |  |  |  |  |  |  |  |  |  |  |  |  |  |  |  |  |  |  |
| *Wu 2023 (68)* |  |  |  |  |  |  |  |  |  |  |  |  |  |  |  |  |  |  |  |  |  | 🗸 |  |  |  |  |  |  |  |  |  |  |  |  | 🗸 |  |  |  |  |  |  |  |  |
| *Xu 2016 (69)* |  |  |  |  |  |  |  |  |  |  |  |  |  |  |  |  |  |  |  |  |  |  |  |  |  |  |  | 🗸 |  |  |  | 🗸 |  |  |  |  |  |  |  |  |  |  |  |
| *Yang 2012 (70)* |  |  |  |  |  |  |  |  |  |  |  |  |  |  |  | 🗸 |  |  |  |  |  |  |  |  |  |  |  |  |  |  |  |  |  |  |  |  |  |  |  |  |  |  |  |
| *Ye 2012 (71)* |  |  |  |  |  |  |  |  |  |  |  |  | 🗸 |  |  | 🗸 |  |  |  |  |  |  |  |  |  |  | 🗸 |  |  |  |  |  | 🗸 |  |  |  |  |  |  |  |  |  |  |
| *Young 2013 (83)* |  |  |  |  |  |  |  |  |  |  |  |  |  |  |  | 🗸^2^ |  |  |  |  |  |  |  |  |  |  |  |  |  |  |  |  |  |  | 🗸^4^ | 🗸^4^ |  |  |  |  |  |  |  |
| *Zandifar 2020 (72)* |  |  |  |  |  |  |  |  |  |  |  |  |  |  |  | 🗸 |  |  |  |  |  |  |  |  |  |  | 🗸 |  |  |  |  |  |  |  |  |  |  |  |  |  |  |  |  |
| *Total* | 7 | 3 | 1 | 1 | 1 | 2 | 1 | 1 | 1 | 1 |  | 1 | 2 | 1 | 1 | 35 | 3 | 1 | 1 | 3 | 2 | 1 | 1 |  | 1 | 1 | 10 | 7 | 1 | 2 | 2 | 2 | 5 | 1 | 4 | 7 | 3 | 1 | 1 | 1 | 1 | 1 |  |

Superscript numbers indicate the number of predictors extracted from this source.

| **Supplementary figure 1C continued.** Predictors per source: MRI. | | | | | | | | | | | | | | | | | | | | | | | | | | | | | | | | |
| --- | --- | --- | --- | --- | --- | --- | --- | --- | --- | --- | --- | --- | --- | --- | --- | --- | --- | --- | --- | --- | --- | --- | --- | --- | --- | --- | --- | --- | --- | --- | --- | --- |
|  | *Retroslenial cortex* | *Frontal Pole* | *Temporal pole* | *Lingual gyrus* | *Supramarginal gyrus* | *Isthmus cingulate cortex* | *Cingulate cortex* | *Orbitofrontal gyrus* | *Orbitofrontal cortex* | *MBL* | *Occipital Gyrus* | *Occipital cortex* | *Fusiform cortex* | *Fusiform gyrus* | *Caudate* | *Putamen* | *Precuneus* | *Cuneus* | *Amygdala* | *Parahippocampal Gyrus* | *Ventricles* | *Fornix/stria terminalis* | *Cingulum* | *Ucinate fasciculus* | *White matter* | *Tensor-based morphometry* | *Voxel-based morphometry* | *Global Grading Biomarker* | *Frontal gyrus* | *Frontal cortex* | *Parahippocampal cortex* | *Pars Triangularis cortex* |
| **Source** | **MRI continued** | | | | | | | | | | | | | | | | | | | | | | | | | | | | | | | |
| *Ardekani 2016 (27)* |  |  |  |  |  |  |  |  |  |  |  |  |  |  |  |  |  |  |  |  |  |  |  |  |  |  |  |  |  |  |  |  |
| *Bapat 2024 (28)* |  |  |  |  |  |  |  |  |  |  |  |  |  |  |  |  |  |  |  |  |  |  |  |  |  |  |  |  |  |  |  |  |
| *Barnes 2014 (29)* |  |  |  |  |  |  |  |  |  |  |  |  |  |  |  |  |  |  |  |  |  |  |  |  |  |  |  |  |  |  |  |  |
| *Cai 2023 (32)* |  |  |  |  |  |  |  |  |  |  |  |  |  |  |  |  |  |  | 🗸 |  |  |  |  |  |  |  |  |  |  |  |  |  |
| *Cui 2011 (73)* | 🗸 |  |  |  |  |  |  |  |  |  |  |  |  |  |  |  |  |  |  |  |  |  |  |  |  |  |  |  |  |  |  |  |
| *Devenand 2008 (36)* |  |  |  |  |  |  |  |  |  |  |  |  |  |  |  |  |  |  |  |  |  |  |  |  |  |  |  |  |  |  |  |  |
| *Devenand 2012 (84)* |  |  |  |  |  |  |  |  |  |  |  |  |  |  |  |  |  |  |  |  |  |  |  |  |  |  |  |  |  |  |  |  |
| *Dobromsylin 2022 (37)* |  |  |  |  |  |  |  |  |  |  |  |  |  |  |  |  |  |  |  |  |  |  |  |  |  |  |  |  |  |  |  |  |
| *Dukart 2015 (74)* |  |  |  |  |  |  |  |  |  |  |  |  |  |  |  |  |  |  |  |  |  |  |  |  |  |  |  |  |  |  |  |  |
| *El-Sappagh 2021 (38)* |  |  |  |  |  |  |  |  |  |  |  |  |  |  |  |  |  |  |  |  |  |  |  |  |  |  |  |  |  |  |  |  |
| *Ezzati 2019 (75)* |  |  |  |  |  |  |  |  |  |  |  |  |  |  |  |  |  |  |  |  |  |  |  |  |  |  |  |  |  |  |  |  |
| *Goel 2023 (40)* |  |  |  |  |  |  |  |  |  |  |  |  |  |  |  |  |  |  |  |  |  | 🗸 | 🗸 | 🗸 | 🗸 |  |  |  |  |  |  |  |
| *Hall 2015 (1) (42)* |  |  |  |  |  |  |  |  |  | 🗸 |  |  |  |  |  |  |  |  | 🗸 | 🗸 | 🗸 |  |  |  |  |  |  |  |  |  |  |  |
| *Hall 2015 (2) (76)* |  |  |  |  |  |  |  |  |  |  |  |  |  |  |  |  |  |  |  |  |  |  |  |  |  | 🗸 | 🗸 |  |  |  |  |  |
| *Hou 2023 (43)* |  |  |  |  |  |  |  |  |  |  |  |  |  |  |  |  |  |  |  |  |  |  |  |  |  |  |  |  |  |  |  |  |
| *Kauppi 2018 (45)* |  |  |  |  |  | 🗸 |  | 🗸 |  |  |  |  |  |  |  |  |  |  |  |  |  |  |  |  |  |  |  |  |  |  |  |  |
| *Khajephiri 2022 (46)* |  |  |  |  |  |  |  |  |  |  |  |  |  |  |  |  |  |  |  |  |  |  |  |  |  |  |  |  |  |  |  |  |
| *Korolev 2016 (47)* |  |  |  |  |  |  |  |  |  |  |  |  |  |  |  |  |  |  |  |  |  |  |  |  |  |  |  |  |  |  |  |  |
| *Lee 2019 (49)* |  |  |  |  |  |  |  |  |  |  |  |  |  |  |  |  |  |  |  |  |  |  |  |  |  |  |  |  |  |  |  |  |
| *Liu 2013 (85)* |  |  |  |  |  |  |  |  |  |  |  |  |  |  |  |  |  |  |  |  |  |  |  |  |  | 🗸 | 🗸 |  |  |  |  |  |
| *Luk 2018 (50)* |  |  |  |  |  |  |  |  |  |  |  |  |  |  |  |  |  |  |  |  | 🗸 |  |  |  |  |  |  |  |  |  |  |  |
| *Mattila 2012 (51)* |  |  |  |  |  |  |  |  |  |  |  |  |  |  |  |  |  |  |  |  |  |  |  |  |  | 🗸 | 🗸 |  |  |  |  |  |
| *Mubeen 2017 (52)* |  |  |  |  |  |  |  |  |  |  |  |  |  |  |  |  |  |  |  |  |  |  |  |  |  |  |  |  |  |  |  |  |
| *Munoz-Ruiz 2014 (53)* |  |  |  |  |  |  |  |  |  |  |  |  |  |  |  |  |  |  |  |  |  |  |  |  |  |  |  |  |  |  |  |  |
| *Ning 2018 (78)* |  |  |  |  |  |  | 🗸^2^ |  |  |  | 🗸 |  | 🗸^2^ |  | 🗸^2^ |  | 🗸^2^ |  | 🗸^2^ | 🗸 | 🗸^2^ |  |  |  |  |  |  |  |  |  |  |  |
| *Pang 2023 (54)* |  |  |  |  |  |  |  |  |  |  |  |  |  |  |  |  |  |  |  |  |  |  |  |  |  |  |  |  |  |  |  |  |
| *Park 2022 (55)* |  |  |  |  |  |  |  |  |  |  |  |  |  |  |  |  |  |  |  |  |  |  |  |  |  |  |  |  |  |  |  |  |
| *Platero 2020 (57)* |  |  |  |  |  |  |  |  |  |  |  |  |  |  |  |  |  |  |  |  |  |  |  |  |  |  |  |  |  |  |  |  |
| *Platero 2021 (58)* |  |  |  |  |  |  |  |  |  |  |  |  |  |  |  |  |  |  |  |  |  |  |  |  |  |  |  |  |  |  |  |  |
| *Rhodius-Meester 2016 (86)* |  |  |  |  |  |  |  |  |  |  |  |  |  |  |  |  |  |  |  |  |  |  |  |  |  | 🗸 | 🗸 |  |  |  |  |  |
| *Runtti 2014 (59)* |  |  |  |  |  |  |  |  |  |  |  |  |  | 🗸^2^ |  |  |  |  |  |  | 🗸^3^ |  |  |  |  |  |  |  |  |  |  |  |
| *Shu 2021 (60)* |  |  |  |  |  |  |  |  |  |  |  |  |  |  |  |  |  |  |  |  |  |  |  |  |  |  |  |  |  |  |  |  |
| *Tabatabaei-Jafari 2018 (61)* |  |  |  |  |  |  |  |  |  |  |  |  |  |  |  |  |  |  |  |  |  |  |  |  |  |  |  |  |  |  |  |  |
| *Tam 2019 (62)* |  |  |  |  |  |  |  |  |  |  |  |  |  |  |  |  |  |  |  |  |  |  |  |  |  |  | 🗸 |  |  |  |  |  |
| *Tang 2021 (63)* |  |  |  |  |  |  |  |  |  |  |  |  |  |  |  |  |  |  |  |  |  |  |  |  |  |  |  |  |  |  |  |  |
| *Tong 2017 (79)* |  |  |  |  |  |  |  |  |  |  |  |  |  |  |  |  |  |  |  |  |  |  |  |  |  |  |  | 🗸 |  |  |  |  |
| *van Maurik 2017 (80)* |  |  |  |  |  |  |  |  |  |  |  |  |  |  |  |  |  |  |  |  |  |  |  |  |  |  |  |  |  |  |  |  |
| *van Maurik 2019 (1) (81)* |  |  |  |  |  |  |  |  |  |  |  |  |  |  |  |  |  |  |  |  |  |  |  |  |  |  |  |  |  |  |  |  |
| *van Maurik 2019 (2) (87)* |  |  |  |  |  |  |  |  |  |  |  |  |  |  |  |  |  |  |  |  |  |  |  |  |  |  |  |  |  |  |  |  |
| *Varatharajah 2019 (64)* |  |  |  |  |  | 🗸 | 🗸 |  | 🗸 |  |  |  |  |  |  |  |  |  |  |  |  |  |  |  |  |  |  |  |  | 🗸 | 🗸 | 🗸 |
| *Wang 2016 (65)* |  |  |  |  |  |  |  |  |  |  |  |  |  |  |  |  |  |  |  |  |  |  |  |  |  |  |  |  |  |  |  |  |
| *Wang 2023 (66)* |  |  |  |  |  |  |  |  |  |  |  |  |  |  |  |  |  |  |  |  |  |  |  |  |  |  |  |  |  |  |  |  |
| *Westman 2012 (82)* |  | 🗸^2^ | 🗸^2^ | 🗸^2^ | 🗸^2^ | 🗸^2^ | 🗸^2^ |  | 🗸^2^ |  |  | 🗸^2^ |  | 🗸^2^ | 🗸^2^ | 🗸^2^ | 🗸^2^ | 🗸^2^ | 🗸^2^ | 🗸^2^ | 🗸^2^ |  |  |  |  |  |  |  | 🗸^2^ |  |  |  |
| *Willette 2014 (67)* |  |  |  |  |  |  |  |  |  |  |  |  |  |  |  |  |  |  |  |  |  |  |  |  |  |  |  |  |  |  |  |  |
| *Wu 2023 (68)* |  |  |  |  |  |  |  |  |  |  |  |  |  | 🗸 |  |  |  |  |  |  |  |  |  |  |  |  |  |  |  |  |  |  |
| *Xu 2016 (69)* |  |  |  |  |  |  |  |  |  |  |  |  |  |  |  |  |  | 🗸 |  |  |  |  |  |  |  |  |  |  |  |  |  |  |
| *Yang 2012 (70)* |  |  |  |  |  |  |  |  |  |  |  |  |  |  |  |  |  |  |  |  | 🗸 |  |  |  |  |  |  |  |  |  |  |  |
| *Ye 2012 (71)* |  |  | 🗸 |  |  |  | 🗸 |  |  |  |  |  |  |  |  |  |  | 🗸 |  |  |  |  |  |  |  |  |  |  |  |  |  |  |
| *Young 2013 (83)* |  |  |  |  |  |  |  |  |  |  |  |  |  | 🗸^2^ |  |  |  |  | 🗸^2^ | 🗸^2^ |  |  | 🗸^2^ |  |  |  |  |  |  |  |  |  |
| *Zandifar 2020 (72)* |  |  |  |  |  |  |  |  |  |  |  |  |  |  |  |  |  |  |  |  |  |  |  |  |  |  |  |  |  |  |  |  |
| *Total* | 1 | 1 | 2 | 1 | 1 | 3 | 4 | 1 | 2 | 1 | 1 | 1 | 1 | 4 | 2 | 1 | 2 | 3 | 5 | 4 | 6 | 1 | 2 | 1 | 1 | 4 | 5 | 1 | 1 | 1 | 1 | 1 |

Superscript numbers indicate the number of predictors extracted from this source.
