# Supplementary material for: Limited generalizability and high risk of bias in multivariable models predicting conversion risk from mild cognitive impairment to dementia: A systematic review
Source: Alzheimers Dement. 2025 Apr 6;21(4):e70069. doi: 10.1002/alz.70069 (PMC11972987; doi:10.1002/alz.70069)
Supplement: Supplementary file 5 — Supporting Information [file ALZ-21-e70069-s011.docx]

| **Supplementary figure 1D.** Predictors per source: PET. | | | | | | | | | | | | | | | | | | | | | | | | | |
| --- | --- | --- | --- | --- | --- | --- | --- | --- | --- | --- | --- | --- | --- | --- | --- | --- | --- | --- | --- | --- | --- | --- | --- | --- | --- |
|  | *PET - general* | *PET - psychoradiomics signature* | *Amyloid-PET* | *FDG-PET* | *AV45-PET* | *Florabetir-PET - cerebellum* | *Florabetir-PET - Amygdala* | *Florabetir-PET - Putamen* | *Florabetir-PET - Superior frontal* | *Florabetir-PET - Thalamus* | *FDG-PET - Global cerebrum* | *FDG-PET - Angular* | *FDG-PET - Temporal* | *FDG-PET - Cingulum* | *FDG-PET - Angular gyrus* | *FDG-PET - Temporal cingulate* | *FDG-PET - Posterior cingulate* | *FDG-PET - Posterior cingulum* | *FDG-PET - Precentral* | *FDG-PET - Postcentral* | *FDG-PET - Posterior cingulum* | *FDG-PET - Superior parietal* | *FDG-PET - Middle temporal* | *PIB-PET - Composite refnorm* | *PIB-PET - Temporal* |
| **Source** | **PET** | | | | | | | | | | | | | | | | | | | | | | | | |
| *Blazhenets 2020 (30)* |  |  | 🗸 | 🗸 |  |  |  |  |  |  |  |  |  |  |  |  |  |  |  |  |  |  |  |  |  |
| *Bouallègue 2017 (31)* |  |  | 🗸^8^ | 🗸^8^ |  |  |  |  |  |  |  |  |  |  |  |  |  |  |  |  |  |  |  |  |  |
| *Cao 2023 (33)* |  |  |  | 🗸 |  |  |  |  |  |  |  |  |  |  |  |  |  |  |  |  |  |  |  |  |  |
| *Dukart 2015 (74)* |  |  |  |  | 🗸 |  |  |  |  |  |  |  |  |  |  |  |  |  |  |  |  |  |  |  |  |
| *El-Sappagh 2021 (38)* | 🗸^3^ |  |  |  |  |  |  |  |  |  |  |  |  |  |  |  |  |  |  |  |  |  |  |  |  |
| *Munoz-Ruiz 2014 (53)* |  |  |  | 🗸 |  |  |  |  |  |  |  |  |  |  |  |  |  |  |  |  |  |  |  |  |  |
| *Pang 2023 (54)* |  |  | 🗸 | 🗸 |  |  |  |  |  |  |  |  |  |  |  |  |  |  |  |  |  |  |  |  |  |
| *Peng 2023 (56)* |  | 🗸 |  |  |  |  |  |  |  |  |  |  |  |  |  |  |  |  |  |  |  |  |  |  |  |
| *van Maurik 2019a (81)* |  |  | 🗸 |  |  |  |  |  |  |  |  |  |  |  |  |  |  |  |  |  |  |  |  |  |  |
| *Varatharajah 2019 (64)* |  |  |  |  |  |  |  |  |  |  |  | 🗸^L^ | 🗸^B^ | 🗸^B^ |  |  |  |  |  |  |  |  |  | 🗸 | 🗸 |
| *Wang 2016 (65)* |  |  |  |  |  | 🗸 |  |  |  |  | 🗸 |  |  |  |  |  |  |  |  |  |  |  |  |  |  |
| *Wang 2023 (66)* |  |  |  |  |  |  |  |  |  |  |  |  |  |  | 🗸 | 🗸 | 🗸 |  |  |  |  |  |  |  |  |
| *Xu 2016 (69)* |  |  |  |  |  |  | 🗸^B^ | 🗸^B^ | 🗸^L^ | 🗸^R^ |  |  |  |  |  |  |  | 🗸^L^ | 🗸^R^ | 🗸^R^ | 🗸^R^ | 🗸^R^ | 🗸^L^ |  |  |
| *Young 2013 (83)* |  |  |  | 🗸 |  |  |  |  |  |  |  |  |  |  |  |  |  |  |  |  |  |  |  |  |  |
| *Total* | 1 | 1 | 4 | 6 | 1 | 1 | 1 | 1 | 1 | 1 | 1 | 1 | 1 | 1 | 1 | 1 | 1 | 1 | 1 | 1 | 1 | 1 | 1 | 1 | 1 |

Superscript numbers indicate the number of predictors extracted from this source; ^L^ left; ^R^ right; ^B^ bilateral.
