# Supplementary material for: Limited generalizability and high risk of bias in multivariable models predicting conversion risk from mild cognitive impairment to dementia: A systematic review
Source: Alzheimers Dement. 2025 Apr 6;21(4):e70069. doi: 10.1002/alz.70069 (PMC11972987; doi:10.1002/alz.70069)
Supplement: Supplementary file 6 — Supporting Information [file ALZ-21-e70069-s009.docx]

| **Supplementary figure 1E.** Predictors per source: fluid biomarkers. | | | | | | | | | | |
| --- | --- | --- | --- | --- | --- | --- | --- | --- | --- | --- |
|  | *CSF-Value* | *CSF-profile* | *Ttau* | *Ptau* | *Amyloid Beta* | *Ttau/Amyloid beta ratio* | *Ptau 181* | *Ptau181/Amyloid beta ratio* |  |  |
| **Source** | **Fluid biomakers** | | | | | | | |  |  |
| *Cui 2011 (73)* |  |  |  |  |  | 🗸 |  | 🗸 |  |  |
| *Franciotti 2023 (39)* |  |  | 🗸 | 🗸 | 🗸 |  |  | 🗸 |  |  |
| *Hall 2015 (1) (42)* | 🗸 | 🗸 | 🗸 | 🗸 | 🗸 |  |  |  |  |  |
| *Hou 2023 (43)* |  |  | 🗸 | 🗸 | 🗸 |  |  |  |  |  |
| *Kruczyk 2012 (77)* |  |  | 🗸 | 🗸 | 🗸 |  |  |  |  |  |
| *Lee 2019 (49)* | ^*^ |  |  |  |  |  |  |  |  |  |
| *Liu 2013 (85)* |  |  | 🗸 |  | 🗸 |  |  |  |  |  |
| *Mattila 2012 (51)* |  |  | 🗸 |  | 🗸 |  |  |  |  |  |
| *Munoz-Ruiz 2014 (53)* |  |  | 🗸 |  | 🗸 |  |  |  |  |  |
| *Pang 2023 (54)* |  |  |  |  | 🗸 |  |  |  |  |  |
| *Rhodius-Meester 2016 (86)* |  |  | 🗸 |  | 🗸 |  |  |  |  |  |
| *Runtti 2014 (59)* |  |  | 🗸 |  | 🗸 |  |  |  |  |  |
| *Tang 2021 (63)* |  |  | 🗸 | 🗸 | 🗸 |  |  |  |  |  |
| *van Maurik 2017 (80)* |  |  | 🗸 |  | 🗸 |  |  |  |  |  |
| *van Maurik 2019b (87)* |  |  |  | 🗸 | 🗸 |  |  |  |  |  |
| *van Maurik 2019b -validation (87)* |  |  | 🗸 |  | 🗸 |  |  |  |  |  |
| *Varatharajah 2019 (64)* |  |  | 🗸 | 🗸 | 🗸 |  |  |  |  |  |
| *Wang 2023 (66)* |  |  | 🗸 |  |  |  | 🗸 |  |  |  |
| *Westman 2012 (82)* |  |  | 🗸 | 🗸 |  |  |  |  |  |  |
| *Yang 2012 (70)* |  |  | 🗸 | 🗸 |  |  |  |  |  |  |
| *Total* | 1 | 1 | 16 | 9 | 15 | 1 | 1 | 2 |  |  |

^*^5 CSF features, not further specified.
