# Supplementary material for: Limited generalizability and high risk of bias in multivariable models predicting conversion risk from mild cognitive impairment to dementia: A systematic review
Source: Alzheimers Dement. 2025 Apr 6;21(4):e70069. doi: 10.1002/alz.70069 (PMC11972987; doi:10.1002/alz.70069)
Supplement: Supplementary file 7 — Supporting Information [file ALZ-21-e70069-s004.docx]

**Supplementary table 1.** Search strategy for PubMed and Embase.

| Subject | Search terms PubMed | Search terms Embase |
| --- | --- | --- |
| MCI | "Cognitive Dysfunction"[Mesh:NoExp]  mild cognitive impairment[Title/Abstract]  MCI[Title/Abstract]  Dementia prodrome[Title/Abstract]  Prodromal dementia[Title/Abstract]  Prodromal Alzheimer*[Title/Abstract] | mild cognitive impairment/  mild cognitive impairment.ti,ab,kf.  MCI.ti,ab,kf.  Dementia prodrome.ti,ab,kf.  Prodromal dementia.ti,ab,kf.  Prodromal Alzheimer*.ti,ab,kf. |
| Dementia | dementia[MeSH Terms]  dementia[Title/Abstract]  Alzheimer*[Title/Abstract]  Lewy Body Disease[Title/Abstract] | exp dementia/  dementia.ti,ab,kf.  Alzheimer*.ti,ab,kf.  Lewy Body Disease.ti,ab,kf. |
| Risk prediction | "Prognosis"[Mesh:NoExp]  Dementia/diagnosis[MeSH Terms]  "Risk Assessment"[Mesh:NoExp]  disease progression[MeSH Terms]  predict*[Title/Abstract]  Risk Assessment[Title/Abstract]  prognos*[Title/Abstract]  progress*[Title/Abstract]  "dementia/diagnostic imaging"[MeSH Terms]  "cognitive dysfunction/diagnostic imaging"[MeSH Terms] | prognosis/  Prognostic assessment/  exp dementia/di  risk assessment/  disease exacerbation/  Disease course/  predict*.ti,ab,kf.  prediction/  Risk assessment.ti,ab,kf.  Prognos*.ti,ab,kf.  Progress*.ti,ab,kf. |
| Predictive performance | predictive value of tests[MeSH Terms]  sensitivity and specificity[MeSH Terms]  prognostic performance[Title/Abstract]  Prediction performance[Title/Abstract]  Predictive performance[Title/Abstract]  accuracy[Title/Abstract]  sensitivity[Title/Abstract]  specificity[Title/Abstract]  "area under the curve"[Title/Abstract]  “Area Under Curve”[Mesh]  AUC[Title/Abstract]  ROC curve[Mesh]  ROC[Title/Abstract]  Receiver operating characteristic*[Title/Abstract]  c-statistic*[Title/Abstract]  c-index[Title/Abstract]  Concordance statistic[Title/Abstract]  Concordance index[Title/Abstract]  Harrell’s C[Title/Abstract]  discriminat*[Title/Abstract]  R2[Title/Abstract]  Brier[Title/Abstract]  calibrat*[Title/Abstract]  positive predictive value[Title/Abstract]  negative predictive value[Title/Abstract]  PPV[Title/Abstract]  NPV[Title/Abstract]  "net benefit"[Title/Abstract]  "decision curve"[Title/Abstract] | predictive value/  Predictive validity/  "sensitivity and specificity"/  Prognostic performance.ti,ab,kf.  Prediction performance.ti,ab,kf.  Predictive performance.ti,ab,kf.  accuracy.ti,ab,kf.  accuracy/  Diagnostic accuracy/  Diagnostic test accuracy study/  sensitivity.ti,ab,kf.  specificity.ti,ab,kf.  area under the curve.ti,ab,kf.  area under the curve/  AUC.ti,ab,kf.  receiver operating characteristic/  ROC.ti,ab,kf.  receiver operating characteristic*.ti,ab,kf.  c-statistic*.ti,ab,kf.  c-index.ti,ab,kf.  Concordance statistic.ti,ab,kf.  Concordance index.ti,ab,kf.  Harrell* C.ti,ab,kf.  Discriminat*.ti,ab,kf.  R2.ti,ab,kf.  Brier.ti,ab,kf.  Calibrat*.ti,ab,kf.  Positive predictive value.ti,ab,kf.  Negative predictive value.ti,ab,kf.  PPV.ti,ab,kf.  NPV.ti,ab,kf.  Net benefit.ti,ab,kf.  Decision curve.ti,ab,kf. |

Search terms within each category (MCI, dementia, risk prediction, and predictive performance) are combined with OR. Categories are combined with AND.
