# Supplementary material for: Limited generalizability and high risk of bias in multivariable models predicting conversion risk from mild cognitive impairment to dementia: A systematic review
Source: Alzheimers Dement. 2025 Apr 6;21(4):e70069. doi: 10.1002/alz.70069 (PMC11972987; doi:10.1002/alz.70069)
Supplement: Supplementary file 8 — Supporting Information [file ALZ-21-e70069-s002.docx]

**Supplementary table 2.** MCI criteria used in the included prediction model studies.

| Criteria | Values |
| --- | --- |
| ADNI criteria | MMSE scores between 24-30 (inclusive), a memory complaint, objective memory loss measured by education adjusted scores on Wechsler Memory Scale 7 Logical Memory II, a CDR of 0.5, absence of significant levels of impairment in other cognitive domains, essentially preserved activities of daily living, and an absence of dementia. |
| Petersen criteria | (1) Memory complaint, preferably corroborated by an informant; (2) objective memory impairment adjusted for age and education; (3) preservation of general cognitive functioning; (4) no or minimal impairment of daily life activities; and (5) not fulfilling the DSM-IIIR (Diagnostic and Statistical Manual of Mental Disorders, 3rd edition, revised) criteria of dementia |
| NIA-AA criteria | (1) subjective memory complaints by participants or caregivers; (2) objective memory decline below –1.0, standard deviation (SD) on either verbal or visual memory tests; (3) normal activities of daily living (ADL), as judged clinically; and (4) not demented. |
| Mayo clinic criteria | (1) memory complaint by patient, family, or physician; (2) normal activities of daily living; (3) normal global cognitive function; (4) objective impairment in memory or in one other area of cognitive function as evident by scores >1.5 S.D. below the age-appropriate mean; (5) CDR score of 0.5; and (6) absence of dementia. |
| AddNeuroMed criteria | (1) memory complaint by patient, family, or physician; (2) normal activities of daily living; (3) MMSE score range between 24 and 30; (4) Geriatric Depression Scale score less than or equal to 5; (5) subject aged 65 years or above; (6) CDR memory score of 0.5 or 1; and (7) absence of dementia according to the NINCDS-ADRDA criteria. |
| NACC criteria | Concern about decline in cognition (e.g. based on input from participant, co-participant, and/or the clinician’s judgment, CDR SB 0.5+, etc); Impairment in one or more cognitive domains, compared to participant’s estimated prior level of lifelong or usual cognitive function, or supported by objective longitudinal neuropsychological evidence of decline; Largely preserved functional independence OR functional dependence that is not related to cognitive decline (e.g., based on clinical judgment) |
