# Supplementary material for: Limited generalizability and high risk of bias in multivariable models predicting conversion risk from mild cognitive impairment to dementia: A systematic review
Source: Alzheimers Dement. 2025 Apr 6;21(4):e70069. doi: 10.1002/alz.70069 (PMC11972987; doi:10.1002/alz.70069)
Supplement: Supplementary file 9 — Supporting Information [file ALZ-21-e70069-s007.docx]

**Supplementary table 3.** Definitions used.

| Study | Subjects included other than MCI | Classification |
| --- | --- | --- |
| *Adelson 2023 (26)* | - | Stable MCI vs Converter MCI |
| *Ardekani 2016 (27)* | - | Stable MCI vs Progressive MCI |
| *Bapat 2024 (28)* | - | Stable MCI vs Progressive MCI |
| *Barnes 2014 (29)* | - | Stable MCI vs Progressive MCI |
| *Blazhenets 2020 (30)* | - | Low, medium and high risk group |
| *Bouallègue 2017 (31)* | Normal = subjects rated  normal both at baseline and during follow-up | Stable MCI vs Converter MCI |
| *Cai 2023 (32)* | - | Non-converter MCI vs converter MCI |
| *Cao 2023 (33)* | CN = cognitively normal | Stable MCI vs unstable MCI |
| *Chang 2022 (34)* | - | Converter MCI vs non-converter MCI |
| *Chun 2022 (35)* | - | Converter MCI vs non-converter MCI |
| *Devenand 2008 (36)* | - | Converter MCI vs non-converter MCI |
| *Dobromsylin 2022 (37)* | - | Stable MCI vs Progressive MCI |
| *El-Sappagh 2021 (38)* | CN = cognitively normal | Stable MCI vs Progressive MCI |
| *Franciotti 2023 (39)* | - | Stable MCI vs converter MCI |
| *Goel 2023 (40)* | HC = health control | Stable MCI vs converter MCI |
| *Hall 2015a (42)* | - | Stable MCI vs Progressive MCI |
| *Hou 2023 (43)* | - | Stable MCI vs Progressive MCI |
| *Jang 2017 (44)* | - | Converter MCI vs non-converter MCI |
| *Kauppi 2018 (45)* | - | Stable MCI vs Progressive MCI |
| *Khajehpiri 2022 (46)* | - | Stable MCI vs Progressive MCI |
| *Korolev 2016 (47)* | - | Stable MCI vs Progressive MCI |
| *Lee 2014 (48)* | - | Stable MCI vs Converter MCI |
| *Lee 2019 (49)* | CN = cognitively normal older adult controls | Non-converter MCI vs Converter MCI |
| *Luk 2018 (50)* | NC = normal controls | Non-converter MCI vs Converter MCI |
| *Mattila 2012 (51)* | - | Stable MCI vs Progressive MCI |
| *Mubeen 2017 (52)* | - | Stable MCI vs Progressive MCI |
| *Munoz-Ruiz 2014 (53)* | - | Stable MCI vs Progressive MCI |
| *Pang 2023 (54)* | - | Stable MCI vs converter MCI |
| *Park 2022 (55)* | - | Stable MCI vs Progressive MCI |
| *Peng 2023 (56)* | - | Stable MCI vs Progressive MCI |
| *Platero 2020 (57)* | - | Stable MCI vs Converter MCI |
| *Platero 2021 (58)* | NC = normal control | Stable MCI vs Progressive MCI |
| *Runtti 2014 (59)* | - | Stable MCI vs Progressive MCI |
| *Shu 2021(60)* | - | Stable MCI vs Progressive MCI |
| *Tabatabaei-Jafari 2018 (61)* | CN = cognitively normal | Converter MCI vs stable + reverter MCI |
| *Tam 2019 (62)* | CN = cognitively normal | Stable MCI vs progressive MCI |
| *Tang 2021 (63)* | - | Stable MCI vs progressive MCI |
| *Varatharajah 2019 (64)* | - | MCI non progressor vs MCI progressor |
| *Wang 2016 (65)* | - | MCI converter vs MCI non converter |
| *Wang 2023 (66)* | - | MCI cases that developed dementia or not |
| *Willette 2014 (67)* | CN = cognitively normal:  CDR of 0; MMSE  inclusively between 24 and 30; no evidence of depression; and no memory complaints | Stable MCI vs progressive MCI |
| *Wu 2023 (68)* | - | Stable MCI vs progressive MCI |
| *Xu 2016 (69)* | NC = normal control:  MMSE scores between 24 and 30 (inclusive); a CDR of 0; non-depressed; without MCI; and non-demented | Stable MCI vs progressive MCI |
| *Yang 2012 (70)* | NC = normal control:  MMSE between 24–30; CDR of 0; non-depressed; non-MCI; and nondemented | Stable MCI vs converter MCI |
| *Ye 2012 (71)* | - | MCI non-converters vs MCI converters |
| *Young 2013 (83)* | HC = healthy control | Stable MCI vs converter MCI |
| *Zandifar 2020 (72)* | - | Stable MCI vs progressive MCI |
| *Cui 2011 (73)* | NC = normal control:  MMSE scores between 24 and 30 (inclusive); CDRof 0; and were non depressed; non MCI; and non demented. | MCI non-converter vs MCI converter |
| *Dukart 2015 (74)* | HC = healthy control subjecs | Stable MCI vs Converter MCI |
| *Ezzati 2019 (75)* | CN = cognitively normal | Stable MCI vs Progressive MCI |
| *Grassi 2019 (41)* | - | Converter MCI vs non-converter MCI |
| *Hall 2015b (76)* | - | Stable MCI vs progressive MCI |
| *Kruczyk 2012 (77)* | Controls:  absence of memory complaints or any other cognitive symptoms; preservation of general cognitive functioning; and no active neurological or psychiatric disease | Stable MCI vs Progressive MCI |
| *Ning 2018 (78)* | CN = cognitively normal | Non-progressing vs progressive MCI |
| *Tong 2017 (79)* | NC = normal control | Stable MCI vs progressive MCI |
| *Van Maurik 2017 (80)* | - | Stable MCI vs progressive MCI |
| *Van Maurik 2019a (81)* | - | Stable MCI vs progressive MCI |
| *Westman 2012 (82)* | CTL = healthy control | Stable MCI vs converter MCI |
| *Devenand 2012 (84)* | - | MCI converter vs MCI non-converter |
| *Liu 2013 (85)* | - | MCI converter vs MCI non-converter |
| *Rhodius-Meester 2016 (86)* | - | Stable MCI vs progressive MCI |
| *van Maurik 2019b (87)* | - | Stable MCI vs progressive MCI |
