# Supplementary material for: Limited generalizability and high risk of bias in multivariable models predicting conversion risk from mild cognitive impairment to dementia: A systematic review
Source: Alzheimers Dement. 2025 Apr 6;21(4):e70069. doi: 10.1002/alz.70069 (PMC11972987; doi:10.1002/alz.70069)
Supplement: Supplementary file 10 — Supporting Information [file ALZ-21-e70069-s008.docx]

**Supplementary table 4.** Model development characteristics.

| Source | Predictor types | Model type | Candidate predictors | Predictor selection strategy | Number of predictors in model | Prediction horizon | Model performance measures |
| --- | --- | --- | --- | --- | --- | --- | --- |
| Model development studies | | | | | | | |
| *Adelson 2023 (26)* | Demographics; cognitive scores; family history; comorbidities | Gradient-boosted tree ensemble (XGboost) | 50 | Forward feature selection | 14 | 2 year | AUC = 0.980  ACC = 93.9%  Sens = 0.846  Spec = 1.000  PPV = 1.000  NPV = 0.909 |
| *Ardekani 2016 (27)* | Demographics; genetics; cognitive scores; MRI | Random Forest | 16 | Mean reduction of Gini impurity index | 10 | 3 year | AUC = 0.77  ACC = 72.6%  Sens = 75.6%  Spec = 69.2% |
|  |  |  |  |  |  | 2 year | AUC = 0.83  ACC = 82.3%  Sens = 86.0%  Spec = 78.2% |
| *Bapat 2024 (28)* | Cognitive scores; MRI | Convolutional Neural Network | >15 | Deep learning approach | 15 | 4 year | AUC = 0.864  Balanced ACC = 79.9%  Sens = 0.906  Spec = 0.835 |
|  |  |  |  |  |  | 2.5 year | AUC = 0.915  Balanced ACC = 81.0%  Sens = 0.913  Spec = 0.95 |
| *Barnes 2014 (29)* | Cognitive scores; MRI | Cox proportional hazards model | 50 | Stepwise approach:  1: features within each domain P < 0.20;  2: features P < 0.05 retained | 5 | 3 year | Harrell’s C = 0.78  CC = good calibration |
| *Blazhenets 2020 (30)* | Genetics; cognitive scores; PET | Cox proportional hazards model | >5 | Different combinations tested in separate models | 5 | During follow-up | Harrel's C = 0.87* |
| *Bouallègue 2017 (31)* | Cognitive scores; PET | Support vector machine | 17 | Implicit feature selection | 17 | During follow-up | ACC = 77%  Sens = 74%  Spec = 78%  PPV = 58%  NPV = 88% |
| *Cai 2023 (32)* | Demographics; genetics; cognitive scores; MRI | Explainable Boosting Machine | 21 | Implicit feature selection | 20 | 5 year | AUC = 0.92 |
| *Cao 2023 (33)* | Demographics; cognitive scores; PET | Convolutional Neural Network | >6 | Deep learning approach | 7 | 3 year | AUC = 0.785  Balanced accuracy = 73.3%  Sens = 0.611  Spec = 0.854 |
| *Chang 2022 (34)* | Cognitive scores; MRI | Logistic regression | 50 | Stepwise approach:  1: Grouping predictors per source in latent variables.  2: Adding variables in a stepwise approach. | 10 | 2 year | AUC = 0.75  ACC = 68%  Sens = 0.56  Spec = 0.76 |
| *Chun 2022 (35)* | Demographics; genetics; cognitive scores | Extreme gradient boost | 19 | Stepwise approach:  1: Domain knowledge to remove unnecessary variables.  2: Significance testing.  3: Multicollinearity management. | 15 | 3 year | AUC = 0.852  ACC = 80.7% |
| *Devenand 2008 (36)* | Cognitive scores; MRI | Logistic regression analysis | 8 | Stepwise and backward logistic regression | 6 | 3 year | AUC = 0.948  ACC = 89.7%  Sens (at spec = 90%) = 85.2%  PPV = 80.8%  LR(+) = 12.4  LR(-) = 0.2 |
| *Dobromsylin 2022 (37)* | Genetics; MRI | Logistic regression model | 28 | Sequential backward feature selection | 2 | 2 year | AUC = 0.794 |
| *El-Sappagh 2021 (38)* | Cognitive scores; MRI; PET | Random Forest | 151 | Recursive feature elimination | 6 | 3 year | AUC = 87.08%  ACC = 87.09%  Precision = 88.07%  Recall = 86.08%  F1-score = 87.08% |
| *Franciotti 2023 (39)* | Cognitive scores; fluid biomarkers | eXtreme Gradient Boosting | 56 | RF features selection technique | 11 | 3 year | ACC = 0.89  Sens = 0.76  Spec = 0.97  PPV = 0.95  NPV = 0.85 |
| *Goel 2023 (40)* | Demographics; genetics; cognitive scores; MRI | Random Forest | 17 | Principal component analysis | 12 | 2 year | AUC = 0.901  Balanced accuracy = 89.4%  F1-score = 88.8% |
| *Grassi 2019 (41)* | Demographics; cognitive scores | Weighted rank of multiple machine learning methods | 20 | Bivariate statistical association tests, recursive feature elimination | 14 | 3 year | AUC = 0.88  Balanced ACC = 0.788  Sens = 77.7%  Spec = 79.9%  PPV= 68.3%  NPV = 86.5%  F1-score = 0.727 |
| *Hall 2015a (42)* | Genetics; cognitive scores; MRI; fluid biomarkers | Disease state index | 16 | Omitting features with relevance under 0.1, corresponding to average  sensitivity and specificity < 0.55. | 18 | During follow-up | AUC = 0.77  ACC = 0.71  Sens = 0.72  Spec = 0.70 |
| *Hou 2023 (43)* | Demographics; genetics; cognitive scores; MRI; fluid biomarkers | Least Absolute Shrinkage and Selection Operator Cox regression | 27 | LASSO | 11 | 5 year | AUC = 0.92  Sens = 0.92  Spec = 0.79  PPV = 0.72  NPV = 0.94 |
| *Jang 2017 (44)* | Demographics; genetics | Logistic regression analysis | 8 | Stepwise logistic regression. 1: Variables significant at a 0.25 level entered the model.  2: Variables remaining significant at a 0.15 level stayed in the model​. | 4 | 3 year | c-statistic: 0.82  CC = good calibration |
| *Kauppi 2018 (45)* | Genetics; cognitive scores; MRI | Cox proportional hazards model | 48 | Different combinations tested in separate models | 10 | 3 year | AUC = 0.84  ACC = 78.9%  Sens = 79.9%  Spec = 77.8% |
| *Khajehpiri 2022 (46)* | Demographics; genetics; cognitive scores; MRI | Xgboost proportional hazard | 14 | Implicit feature selection | 12 | During follow-up | C-index = 84.5% |
| *Korolev 2016 (47)* | Cognitive scores; MRI | Probabilistic multiple kernel learning (pMKL) classification | 787 | Combined filter-wrapper approach | 6 | 3 year | AUC = 0.87  ACC = 79.9%  CCC = 0.95  Sens = 83.4%  Spec = 76.4% |
| *Lee 2014 (48)* | Demographics; cognitive scores | Cox regression analysis | 48 | Stepwise approach:  1: Predictors per domain examined using p < 0.20.  2: Features p < 0.05 retained in the final combined predictive model​ | 6 | 3 year | Harrel’s C = 0.71  CC = good calibration |
| *Lee 2019 (49)* | Demographics; genetics; cognitive scores; MRI; fluid biomarkers | Recurrent neural network | 11 | Deep learning approach | 14 | 2 year | ACC = 0.76  Sens = 0.80  Spec = 0.76 |
| *Luk 2018 (50)* | Demographics; genetics; cognitive scores; MRI | Binary logistic regression model | 18 | Stepwise approach:  1: Voxelwise comparisons of features between stable and converter.  2: Combining selected features in logistic regression model. | 5 | 3 year | ACC = 76.2% |
| *Mattila 2012 (51)* | Genetics; cognitive scores; MRI; fluid biomarkers | Disease state index | 141 | Disease state index method (no explicit feature selection) | 19 | 2 year | Sens = 85.4%  Spec = 87.6% |
| *Mubeen 2017 (52)* | Demographics; genetics; cognitive scores; MRI; PET | Random Forest | 18 | Implicit feature selection | 18 | 3 year | AUC = 0.82 ACC = 71.7%  Sens = 69.7%  Spec = 75.3% |
|  |  |  |  |  |  | 2.5 year | AUC = 0.87 ACC = 80.2%  Sens = 79.6%  Spec = 81.2% |
| *Munoz-Ruiz 2014 (53)* | Genetics; cognitive scores; MRI; PET; fluid biomarkers | Disease state index | >10 | Disease state index method (no explicit feature selection) | 19 | 2.5 year | ACC = 0.70  Sens = 0.71  Spec = 0.68 |
| *Pang 2023 (54)* | Cognitive scores; MRI | Random forest | 20 | Univariate selection methods: Information Gain, chi-squared test, Fisher score, and ANOVA.  Embedded methods: LASSO and Decision Trees. | 19 | 2 year | AUC = 0.854  ACC = 0.853  Sens = 0.852  Spec = 0.856 |
| *Park 2022 (55)* | Demographics; genetics; cognitive scores; MRI | LightGBM | 13 | Implicit feature selection | 12 | 2 year | AUC = 0.792  ACC = 0.711  Precision = 0.651  Recall = 0.659  F1 score = 0.655 |
| *Peng 2023 (56)* | Cognitive scores; PET | Integrated machine learning: SVM, naïve Bayes, RF, KNN | 283 | Initial dimensionality reduction of radiomics features, then backward stepwise selection. | 3 | 8 year | AUC = 0.865  Sens = 0.839  Spec = 0.806 |
| *Platero 2020 (57)* | Cognitive scores; MRI | Linear mixed effects model | 26 | Minimal redundancy maximal relevance algorithm | 7 | 3 year | AUC = 0.860  ACC = 77.7%  Sens = 79.2%  Spec = 75.9% |
|  |  |  |  |  |  | 2 year | AUC = 0.908  ACC = 81.6%  Sens = 77.8%  Spec = 84.7% |
| *Platero 2021 (58)* | Demographics; genetics; cognitive scores; MRI | Linear mixed effects model | 51 | Minimum redundancy maximum relevance algorithm for preselecting features, followed by a nested cross-validation approach | 10 | 3 year | AUC = 0.858  ACC = 77.3%  Sens = 84.7%  Spec = 70.5% |
|  |  |  |  |  |  | 2.5 year | AUC = 0.879  ACC = 79.2%  Sens = 84.8%  Spec = 74.0% |
|  |  |  |  |  |  | 2 year | AUC = 0.892  ACC = 80.9%  Sens = 83.8%  Spec = 78.4% |
| *Runtti 2014 (59)* | Genetics; cognitive scores; MRI | Disease state index | 114 | Disease state index method (no explicit feature selection) | 31 | 3 year | AUC = 82.3%  ACC = 76.9%  Sens = 82.2%  Spec = 73.0% |
| *Shu 2021 (60)* | Demographics; genetics; cognitive scores; MRI | Support vector machine | 385 | Dimensionality reduction methods to select MRI features for model development.  During model development: maximum relevance minimum redundancy algorithm, LASSO | 13 | 2 year | AUC = 0.794  CC = good calibration  Sens = 0.672  Spec = 0.798 |
|  |  |  |  |  |  | 3 year | AUC = 0.797  CC = good calibration  Sens = 0.787  Spec = 0.685 |
|  |  |  |  |  |  | 4 year | AUC = 0.742  Sens = 0.75  Spec = 0.82 |
| *Tabatabaei-Jafari 2018 (61)* | Cognitive scores; MRI | Discriminant analysis | 3 | Different combinations tested in separate models | 2 | 5 year | ACC = 74.6  AUC = 0.81  Sens = 78.6%  Spec = 69.5%  PPV = 76.2%  NPV = 72.4%  LR(+) = 2.6  LR (-) = 0.3 |
| *Tam 2019 (62)* | Demographics; cognitive scores; MRI | Linear support vector machine | 16 | Stepwise approach:  1: linear support vector machine (SVM) model to identify patterns of atrophy and cognitive decline, resulting in highly predictive signatures (HPS).  2: logistic regression model with L1 regularization | 10 | 3 year | ACC = 85.1%***  Sens = 47.3%  Spec = 96.7%  PPV = 81.2% |
| *Tang 2021 (63)* | Genetics; cognitive scores; MRI; fluid biomarkers | Cox proportional hazards model | 4874 | Spearman rank correlation to select radiomics features from MRI, LASSO | 48 | 5 year | c-index = 0.907  CC = good calibration |
| *Varatharajah 2019 (64)* | MRI; PET; fluid biomarkers | Support vector machine – linear | 94 | Joint Mutual Information | 22 | 3 year | AUC = 0.93  ACC = 0.81  Sens = 0.93  Spec = 0.77  Precision = 0.64  Recall = 0.93  F1-score = 0.75 |
| *Wang 2016 (65)* | MRI; PET | Partial least squares analysis | >4 | Partial Least Squares | 3 | 3 year | ACC = 86.05%  Sens = 81.25%  Spec = 90.77% |
| *Wang 2023 (66)* | Demographics; MRI; PET; fluid biomarkers | Bayesian Cox regression model | 34 | Informative and noninformative priors. | 12 | 3 year | Harrel’s C = 0.75  ICI = 0.06 |
| *Willette 2014 (67)* | Demographics; cognitive scores; MRI; fluid biomarkers | Discriminant classification analysis | 39 | Stepwise discriminant analysis | 35 | 2 year | AUC = 0.896  ACC = 83.3%  Sens = 76.7%  Spec = 89.1% |
| *Wu 2023 (68)* | Cognitive scores; MRI | Logistic regression | 29 | LASSO | 8 | 3 year | AUC = 0.91  ACC = 0.83  CC = good calibration  Precision = 0.80 |
| *Xu 2016 (69)* | MRI; PET | Weighted multi-modality sparse representation-based classification | 270 | Two-sample t-test | 18 | 3 year | ACC = 82.5%  Sens = 81.5%  Spec = 83.5% |
| *Yang 2012 (70)* | MRI; fluid biomakers | Linear support vector machine | 45 | Implicit feature selection | 5 | 2 year | ACC = 66.7%  Sens = 82%  Spec = 51.4% |
| *Ye 2012 (71)* | Genetics; cognitive scores; MRI | Sparse logistic regression model | 262 | L1-norm regularization | 15 | 4 year | AUC = 0.859 |
| *Zandifar 2020 (72)* | Demographics; cognitive scores; MRI; PET | Naïve Bayes classifier | 11 | Implicit feature selection | 9 | Different time points | ACC = 81.3% |
| Model development and validation studies | | | | | | | |
| *Cui 2011 (73)* | Cognitive scores; MRI; fluid biomakers | Support Vector machine | 342 | Filter and Wrapper approach for MRI and CSF features, filter method for neuropsychological measures. | 13 | 2 year | AUC = 0.796  ACC = 67.13%  Sens = 96.43  Spec = 48.28 |
| *Dukart 2015 (74)* | Cognitive scores; MRI; PET | Naïve Bayes classification | 62 | Bayesian Markov Blanket approach | 7 | 2 year | ACC = 87% |
| *Ezzati 2019 (75)* | Demographics; genetics; MRI | Ensemble linear discriminant model | 51 | Implicit feature selection | 51 | 2 year | ACC = 74.9%  Sens = 71.5%  Spec = 76.3% |
|  |  |  |  |  |  | 3 year | ACC = 75.3%  Sens= 65.2%  Spec = 79.7% |
|  |  |  |  |  |  | 4 year | ACC = 77.0%  Sens = 59.6%  Spec = 86.1% |
| *Hall 2015b (76)* | Demographics; genetics; cognitive scores; MRI | Disease State Index | 8 | Disease state index method (no explicit feature selection) | 5 | During follow-up | AUC = 0.74 |
| *Kruczyk 2012 (77)* | Demographics; genetics; cognitive scores; fluid biomakers | Monte Carlo feature selection (MCFS) and Rosetta for generating rule based models | 7 | Monte Carlo Feature Selection combined with Rosetta. | 5 | 4 year | AUC = 0.92 |
| *Ning 2018 (78)* | Genetics; MRI | Neural network | 36 | Deep learning approach | 52 | 2 year | AUC = 0.835 |
| *Tong 2017 (79)* | Demographics; cognitive scores; MRI | Random forest | >1.8 million* | Elastic Net Regularization | 7 | 3 year | AUC = 87.0%  ACC = 80.7%  Sens = 86.7%  Spec = 72.6% |
| *van Maurik 2017 (80)* | Demograhipcs; cognitive scores; MRI; fluid biomarkers | Cox proportional hazards model | 8 | Backward selection | 5 | 3 year | Internal validation: Harrel’s C = 0.70  External validation:  Harrel’s C = 0.73 |
| *van Maurik 2019a (81)* | Demograhpics; genetics; cogntive scores; MRI; PET | Cox proportional hazards analysis | 7 | Backward selection | 6 | 3 year | Internal validation: Harrel’s C = 0.82  External validation:  Harrel’s C = 0.76 |
| *Van Maurik 2019b (87)* | Demographics; MRI; fluid biomarkers | Cox proportional hazards analysis | 7 | Backward selection | 5 | 3 year | Harrel’s C = 0.74  CC = good calibration |
| *Westman 2012 (82)* | MRI; fluid biomarkers | Orthogonal partial  least squares | 60 | Implicit feature selection | 60 | 2 year | AUC = 0.610  ACC = 66.4%  PPV = 55.1%  NPV = 79.4% |
|  |  |  |  |  |  | 3 year | AUC = 0.578  ACC = 66.1%  PPV = 61.8%  NPV = 70.4% |
| *Young 2013 (83)* | Genetics; MRI; PET | Gaussian Process | 41 | Implicit feature selection | 3 | 3 year | AUC = 0.795  ACC = 69.9%  Balanced ACC = 74.1%  Sens = 78.7%  Spec = 65.6% |
| Model validation studies | | | | | | | |
| *Devenand 2012 (84)* | Demographics; cognitive scores; MRI | Validation QD study (Devenand 2008) | - | - | 7 | 3 year | AUC = 0.865  correct class = 77.01 |
| *Liu 2013 (85)* | Demographics; cognitive scores; MRI; fluid biomarkers | Validation of PredictND tool | - | - | 17 | 3 year | PredictND tool:  ACC = 72%  Sens = 73%  Spec = 71%  Clinician + PredictAD tool:  ACC = 71%  Sens = 75%  Spec = 68% |
| *Rhodius-Meester 2016 (86)* | Demographics; cognitive scores; MRI; fluid biomarkers | Validation of PredictND tool | - | - | 19 | During follow-up | AUC = 0.82  Sens = 0.80  Spec = 0.66  Youden = 0.46 |
| *van Maurik 2019b (87)* | Cognitive scores; fluid biomarkers | Validation of model proposed in Van Maurik 2017 | - | - | 3 | During follow-up | Harrell’s C = 0.74  CC = good calibration |

CC = calibration curve; CCC = concordance correlation coefficient; ICI = integrated calibration index; LASSO: Least Absolute Shrinkage and Selection Operator.

Implicit feature selection: machine learning methods often inherently involve a form of predictor selection, which is implicitly involved as an automatic feature selection method.

*over 1.8 million voxel features from 1 single MRI, the relevant voxel features are combined in a global grading biomarker.
