# Supplementary material for: Limited generalizability and high risk of bias in multivariable models predicting conversion risk from mild cognitive impairment to dementia: A systematic review
Source: Alzheimers Dement. 2025 Apr 6;21(4):e70069. doi: 10.1002/alz.70069 (PMC11972987; doi:10.1002/alz.70069)
Supplement: Supplementary file 11 — Supporting Information [file ALZ-21-e70069-s003.docx]

| **Supplementary table 5.**  PROBAST results per domain per study. | | | | | | | | | | | |
| --- | --- | --- | --- | --- | --- | --- | --- | --- | --- | --- | --- |
| **Source** | **RoB** | | | |  | **Applicability** | | |  | **Overall** | |
|  | **Participants** | **Predictors** | **Outcome** | **Analysis** |  | **Participants** | **Predictors** | **Outcome** |  | **RoB** | **Applicability** |
| *Adelson 2023 (26)* | - | + | - | - |  | + | + | + |  | - | + |
| *Ardekani 2016 (27)* | + | + | - | - |  | + | + | + |  | - | + |
| *Bapat 2024 (28)* | - | + | ? | - |  | + | + | + |  | - | + |
| *Barnes 2014 (29)* | + | + | - | - |  | + | + | + |  | - | + |
| *Blazhenets 2020 (30)* | - | + | ? | - |  | + | + | ? |  | - | + |
| *Bouallègue 2017 (31)* | + | + | ? | - |  | - | + | ? |  | - | - |
| *Cai 2023 (32)* | - | + | - | - |  | + | + | + |  | - | + |
| *Cao 2023 (33)* | - | + | ? | - |  | + | + | + |  | - | + |
| *Chang 2022 (34)* | - | + | ? | - |  | + | + | + |  | - | + |
| *Chun 2022 (35)* | + | + | ? | - |  | + | + | + |  | - | + |
| *Cui 2011 (73)* | - | + | ? | - |  | + | + | + |  | - | + |
| *Devenand 2008 (36)* | + | + | + | - |  | - | + | + |  | - | - |
| *Devenand 2012 (84)* | - | ? | ? | - |  | + | + | ? |  | - | ? |
| *Dobromsylin 2022 (37)* | ? | + | ? | - |  | + | + | + |  | - | + |
| *Dukart 2015-dev (74)* | ? | + | ? | - |  | + | + | ? |  | - | ? |
| *Dukart 2015-val (74)* | - | + | ? | - |  | + | ? | ? |  | - | ? |
| *El-Sappagh 2021 (38)* | - | + | ? | - |  | + | + | + |  | - | + |
| *Ezzati 2019-dev (75)* | - | + | ? | - |  | + | + | + |  | - | + |
| *Ezzatti 2019- val (75)* | - | + | ? | - |  | + | + | + |  | - | + |
| *Franciotti 2023 (39)* | ? | + | ? | - |  | + | + | + |  | - | + |
| *Goel 2023 (40)* | ? | + | ? | - |  | + | + | + |  | - | + |
| *Grassie 2019-dev (41)* | - | + | - | - |  | + | + | + |  | - | + |
| *Grassie 2019-val (41)* | - | + | - | - |  | + | + | + |  | - | + |
| *Hall 2015a (42)* | ? | - | ? | - |  | + | + | ? |  | - | ? |
| *Hall 2015b-dev (76)* | + | - | ? | - |  | + | + | - |  | - | - |
| *Hall 2015b-val (76)* | + | - | ? | - |  | + | + | - |  | - | - |
| *Hou 2023 (43)* | - | + | ? | - |  | + | + | + |  | - | + |
| *Jang 2017 (44)* | - | + | ? | - |  | + | + | + |  | - | + |
| *Kauppi 2018 (45)* | - | ? | ? | - |  | + | + | + |  | - | + |
| *Khajehpiri 2022 (46)* | - | + | - | - |  | + | + | + |  | - | + |
| *Korolev 2016 (47)* | + | + | ? | - |  | + | + | + |  | - | + |
| *Kruczyk 2012 (77)* | ? | ? | ? | - |  | + | + | + |  | - | + |
| *Lee 2014 (48)* | - | + | - | - |  | + | + | + |  | - | + |
| *Lee 2019 (49)* | ? | + | ? | - |  | + | ? | + |  | - | ? |
| *Liu 2013 (85)* | ? | ? | ? | - |  | + | + | + |  | - | + |
| *Luk 2018 (50)* | ? | ? | ? | - |  | + | + | + |  | - | + |
| *Mattila 2012 (51)* | - | + | - | - |  | + | + | + |  | - | + |
| *Mubeen 2017 (52)* | - | + | - | - |  | + | + | + |  | - | + |
| *Munoz-Ruiz 2014 (53)* | ? | + | ? | - |  | + | + | + |  | - | + |
| *Ning 2018-dev (78)* | + | + | ? | - |  | + | + | + |  | - | + |
| *Ning 2018-val (78)* | + | + | ? | - |  | + | + | + |  | - | + |
| *Pang 2023 (54)* | ? | + | ? | - |  | + | + | ? |  | - | + |
| *Park 2022 (55)* | - | + | - | - |  | + | + | + |  | - | + |
| *Peng 2023 (56)* | - | + | ? | - |  | + | + | + |  | - | + |
| *Platero 2020 (57)* | ? | + | ? | - |  | + | + | + |  | - | + |
| *Platero 2021 (58)* | ? | + | ? | - |  | + | + | ? |  | - | ? |
| *Rhodius-Meester 2016 (86)* | - | + | ? | - |  | + | + | ? |  | - | ? |
| *Runtti 2014 (59)* | - | + | ? | - |  | + | + | + |  | - | + |
| *Shu 2021 (60)* | - | + | + | - |  | + | + | ? |  | - | ? |
| *Tabatabaei-Jafari 2018 (61)* | + | + | - | - |  | + | + | + |  | - | + |
| *Tam 2019 (62)* | - | + | ? | - |  | + | + | + |  | - | + |
| *Tang 2021 (63)* | - | + | ? | - |  | + | + | + |  | - | + |
| *Tong 2017 (79)* | ? | + | ? | - |  | + | + | + |  | - | + |
| *van Maurik 2017-dev (80)* | - | + | ? | - |  | + | + | + |  | - | + |
| *van Maurik 2017-val (80)* | ? | ? | ? | - |  | + | + | + |  | - | + |
| *van Maurik 2019a-dev (81)* | - | + | ? | - |  | + | + | + |  | - | + |
| *van Maurik 2019a-val (81)* | ? | - | ? | - |  | ? | + | ? |  | - | ? |
| *van Maurik 2019b-dev (87)* | - | ? | ? | - |  | + | + | ? |  | - | ? |
| *van Maurik 2019b-val (87)* | - | ? | ? | - |  | + | + | ? |  | - | ? |
| *Varatharajah 2019 (64)* | - | + | ? | - |  | + | + | + |  | - | + |
| *Wang 2016 (65)* | - | + | ? | - |  | + | + | + |  | - | + |
| *Wang 2023 (66)* | - | + | ? | - |  | + | + | + |  | - | + |
| *Westman 2012-dev (82)* | - | + | ? | - |  | + | + | + |  | - | + |
| *Westman 2012-val (82)* | - | + | ? | - |  | + | + | + |  | - | + |
| *Willette 2014 (67)* | - | + | ? | - |  | + | + | + |  | - | + |
| *Wu 2023 (68)* | + | + | ? | - |  | + | + | + |  | - | + |
| *Xu 2016 (69)* | ? | + | ? | - |  | + | + | + |  | - | + |
| *Yang 2012 (70)* | - | + | ? | - |  | + | + | + |  | - | + |
| *Ye 2012 (71)* | - | + | ? | - |  | + | + | + |  | - | + |
| *Young 2013 (83)* | - | + | ? | - |  | + | + | + |  | - | + |
| *Zandifar 2020 (72)* | - | + | ? | - |  | + | + | + |  | - | + |

+ Low; - High; ? Unclear; dev = development; val = validation.
